# Conditional Overexpression of *Serpine2* Promotes Hair Cell Regeneration from Lgr5+ Progenitors in the Neonatal Mouse Cochlea

**DOI:** 10.1002/advs.202412653

**Published:** 2025-03-17

**Authors:** Hairong Xiao, Jiheng Wu, Lixuan Huang, Ying Ma, Leilei Wu, Yanqin Lin, Zixuan Ye, Xin Tan, Xujun Tang, Wei Tong, Mingchen Dai, Yintao Wang, Xia Sheng, Renjie Chai, Shasha Zhang

**Affiliations:** ^1^ State Key Laboratory of Digital Medical Engineering Department of Otolaryngology Head and Neck Surgery Zhongda Hospital School of Life Sciences and Technology Advanced Institute for Life and Health Jiangsu Province High‐Tech Key Laboratory for Bio‐Medical Research Southeast University Nanjing 210096 China; ^2^ Southeast University Shenzhen Research Institute Shenzhen 518063 China; ^3^ School of Public Health Tongji Medical College Huazhong University of Science and Technology Wuhan 430030 China; ^4^ Department of Environmental Health School of Environmental Science and Engineering Hainan University Haikou 570228 China; ^5^ Institute for Stem Cells and Regeneration Chinese Academy of Science Beijing 100081 China; ^6^ Department of Otolaryngology Head and Neck Surgery Sichuan Provincial People's Hospital University of Electronic Science and Technology of China Chengdu 610000 China; ^7^ Co‐Innovation Center of Neuroregeneration Nantong University Nantong 226001 China

**Keywords:** hair cells, Lgr5+ progenitors, regeneration, *Serpine2*

## Abstract

Neonatal cochlear Lgr5+ progenitors retain limited hair cells (HCs) regenerative capacity, but the regulatory network remains incompletely defined. Serpin family E member 2 (*Serpine2*) is shown to participate in regulating proliferation and differentiation of cochlear Lgr5+ progenitors in the previous in vitro study. Here, the expression pattern and in vivo roles of *Serpine2* in HC regeneration are explored by transgenic mice. It is found that *Serpine2* is expressed in the mouse cochlea after birth with a downward trend as the mice age. In addition, *Serpine2* conditional overexpression in vivo in Lgr5+ progenitors of neonatal mice cochlea results in an increased number of ectopic HCs in a dose‐dependent manner. *Serpine2* knockdown ex vivo and in vivo can inhibit HC regeneration. EdU assay and lineage tracing assay demonstrate these ectopic HCs likely originate from Lgr5+ progenitors through direct transdifferentiation rather than through mitotic regeneration. Moreover, single‐nucleus RNA sequencing analysis and mRNA level validation reveal that conditionally overexpressed *Serpine2* likely induces HC regeneration via inhibiting sonic hedgehog (SHH) signal pathway and inducing *Atoh1* and *Pou4f3* transcription factor. In brief, these data indicate that *Serpine2* plays a pivotal role in HC regeneration from Lgr5+ progenitors in the neonatal mouse cochlea, and this suggests a new avenue for future research into HC regeneration.

## Graphical abstract

This study indicates that *Serpine2* plays a pivotal role in hair cell (HC) regeneration from Lgr5+ progenitors in the neonatal mouse cochlea. Conditional overexpression of *Serpine2* in cochlear Lgr5+ progenitors results in a significantly increased number of ectopic HCs, particularly ectopic inner HCs, and these HCs are likely derived from Lgr5+ progenitors via direct transdifferentiation rather than mitotic regeneration. Overexpression of *Serpine2* enhances the expression of *Atoh1* and *Pou4f3* transcription factors and reduces the activity of the SHH (sonic hedgehog) signaling pathway, thereby promoting HC regeneration.

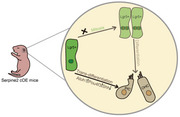

## 1 Introduction

Sensorineural hearing loss (SNHL) is one of the most prevalent health concerns worldwide and is primarily caused by loss of or damage to cochlear hair cells (HCs).^[^ ^1^ ^]^ Loss of HCs in adult mammals is irreversible, but supporting cells (SCs) of fish and birds have the ability to regenerate into HCs.^[^ ^2^ ^]^ Previous research has demonstrated that HCs can be regenerated in the neonatal mouse inner ear, but this ability is extremely transient and is rapidly lost soon after birth.^[^ ^3^ ^]^ Because of the permanence of HC loss in mammals, there remains an urgent need to elucidate the molecular mechanisms of HC regeneration to develop therapeutic interventions for SNHL.

*Lgr5* is a Wnt‐targeted gene that marks not only somatic stem cells in self‐renewing organs, but also progenitor cells in damaged tissue, such as small intestine,^[^ ^4^ ^]^ stomach,^[^ ^5^ ^]^ hair follicle,^[^ ^6^ ^]^ and liver.^[^ ^7^ ^]^ In addition, *Lgr5* can mark cancer stem cells in intestinal adenomas,^[^ ^8^ ^]^ gastric cancers,^[^ ^9^ ^]^ and basal cell carcinomas,^[^ ^10^ ^]^ which are involved in tumorigenesis. The expression of *Lgr5* has been reported in certain types of SCs in the mouse cochlea, such as inner phalangeal cells, third‐row Deiters’ cells, inner pillar cells, and a portion of greater epithelial ridge cells, and have the characteristics of inner ear progenitors.^[^ ^4^, ^11^ ^]^ Cochlear SCs or progenitor cells may proliferate and differentiate in response to multiple signaling pathways, resulting in HC regeneration.^[^ ^12^ ^]^ There are two commonly acknowledged pathways for HC regeneration, namely mitotic regeneration and direct transdifferentiation,^[^ ^12^, ^13^ ^]^ and several critical genes, including *Atoh1*,^[^ ^14^ ^]^ *Gfi1*,^[^ ^15^ ^]^ *Pou4f3*,^[^ ^16^ ^]^ *Foxg1*,^[^ ^17^ ^]^ and other genes, as well as the Wnt,^[^ ^18^ ^]^ Notch,^[^ ^19^ ^]^ and Hippo^[^ ^20^ ^]^ signaling pathways, can regulate the regeneration ability of Lgr5+ progenitors in vivo. In addition, compared to regulating individual factors, coregulation of these genes and signaling pathways has been demonstrated to further promote HC regeneration.^[^ ^21^ ^]^ The coregulation of *Gfi1*, *Pou4f3*, and *Atoh1* enhances the transdifferentiation from SCs to HCs and promotes the further maturation of regenerated HC to a certain extent.^[^ ^21^, ^22^ ^]^ However, HC regeneration, especially in adults, is still very limited, even when multiple genes and signaling pathways are regulated simultaneously.^[^ ^21^, ^23^ ^]^ Therefore, it is necessary to screen novel genes that can effectively promote HC regeneration. In our previous research, we analyzed the transcriptomes of Lgr5+ progenitors under different circumstances and with different capacities for HC regeneration and identified several novel genes that might participate in HC regeneration.^[^ ^24^ ^]^ Of these, we found that serpin family E member 2 (*Serpine2*) seems to have a regulatory effect on HC regeneration in vitro (unpublished data).

*Serpine2*, also referred to as protease nexin‐1, is an important member of the SERine Protease INhibitor (Serpin) superfamily,^[^ ^25^ ^]^ which is expressed in various tissues throughout the body.^[^ ^26^ ^]^ *Serpine2* plays vital roles in many physiological and pathological processes such as embryonic development,^[^ ^27^ ^]^ blood clotting,^[^ ^28^ ^]^ and thrombosis^[^ ^29^ ^]^ via its antiprotease activity. *Serpine2* is also involved in the progression of several human cancers, and it does so by regulating various cellular physiological processes, especially cell proliferation.^[^ ^30^ ^]^ However, whether *Serpine2* takes part in regulating the proliferation and differentiation of cochlear Lgr5+ progenitors during HC regeneration in vivo in the mouse cochlea remains unknown.

Here, we conditionally overexpressed *Serpine2* (*Serpine2* cOE) in Lgr5+ progenitors by transgenic mice, and we observed significant ectopic HCs, particularly inner HCs (IHCs). The EdU assay and lineage tracing assay indicated that these ectopic HCs likely originated from Lgr5+ progenitors through direct transdifferentiation rather than through mitotic regeneration. And *Serpine2* knockdown resulted in a decrease in the number of HCs derived from Lgr5+ progenitors. In addition, both single‐nucleus RNA sequencing (snRNA‐seq) and real‐time quantitative polymerase chain reaction (RT‐qPCR) suggested that *Serpine2* cOE may promote transdifferentiation of newly regenerated HCs by inhibiting the sonic hedgehog (SHH) signaling pathway and inducing *Atoh1* and *Pou4f3* transcription factors. Our results indicate that *Serpine2* is a regulatory gene capable of inducing HC regeneration from Lgr5+ progenitors in the mouse cochlea, suggesting a new line of study for the future clinical therapy of SNHL.

## 2 Results

### 2.1 The Expression of *Serpine2* in Wild‐Type Mice and *Serpine2* cOE Mice

**Figure 1:**
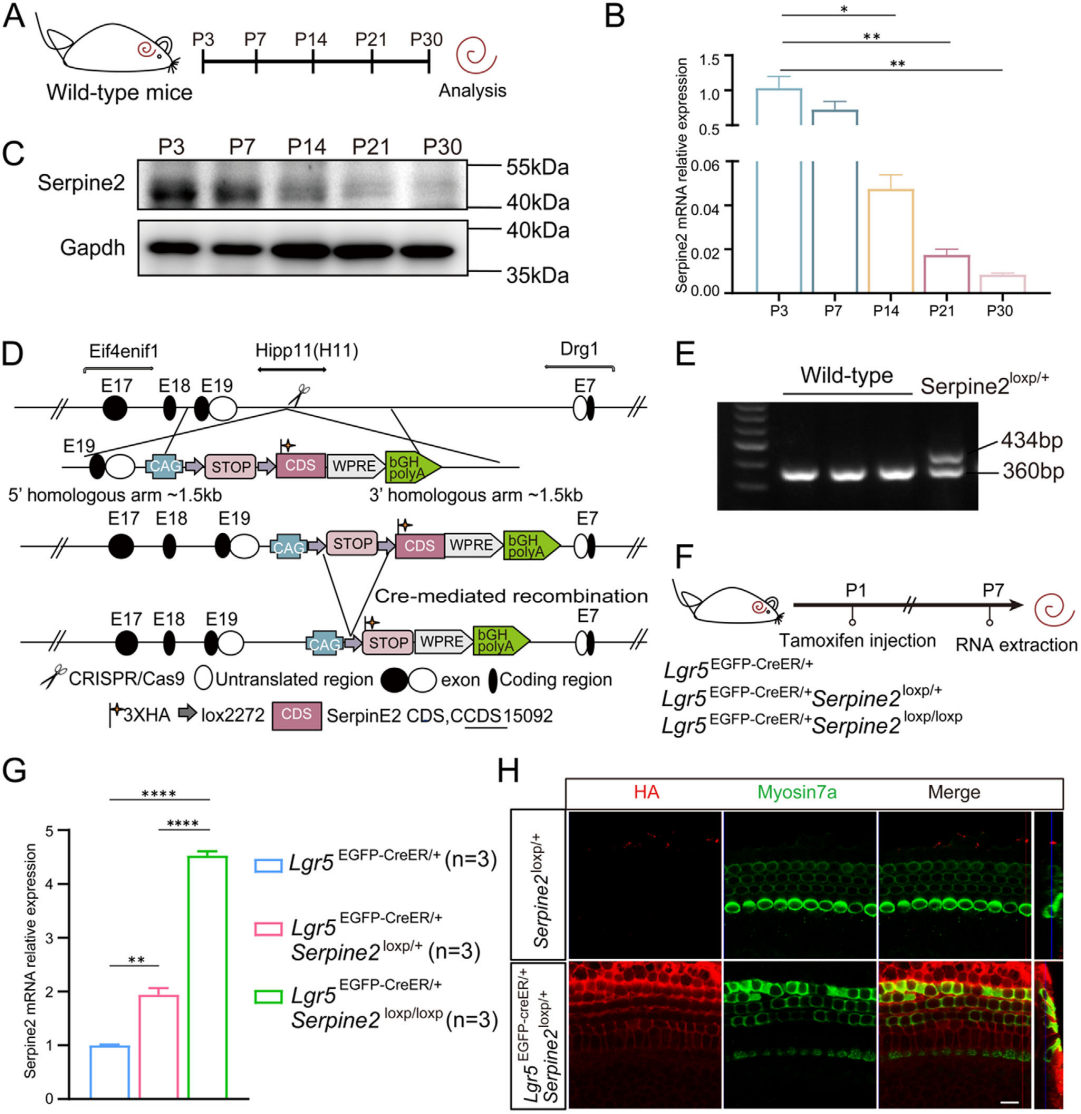
Expression pattern of *Serpine2* in the mouse cochlea and conditional overexpression of *Serpine2* in Lgr5+ progenitors. A) Flow chart of the *Serpine2* expression pattern study in wild‐type mouse cochlea. B,C) *Serpine2* mRNA and protein expression were assessed by RT‐qPCR (B) and western blotting (C). D) Construction of *Serpine2* ^loxp/+^ mice. A vector containing the *Serpine2* gene sequence and a 3 × HA tag was inserted by CRISPR/Cas9 technology at the Hipp11 (H11) site. E) Genotyping of *Serpine2* ^loxp/+^ mice. The sizes of the DNA bands amplified by the wild‐type and mutant alleles were 360 and 434 bp, respectively. F) Flow chart of the *Serpine2* expression pattern study in *Serpine2* cOE mouse cochlea. (G,H) The *Serpine2* expression in *Serpine2* cOE mice and control mice was detected by RT‐qPCR (G) and immunofluorescence staining (H). *Serpine2* expression was detected by HA tag staining (red). Myosin7a (green) was used as the HC marker. “*n*” refers to the number of mice used. Scale bars are 20 µm in (H). *p < 0.05, **p < 0.01, ****p < 0.0001.

We first investigated the *Serpine2* expression pattern during cochlear development after birth in wild‐type mice. RNA and protein were extracted from the cochleae at different ages (postnatal day (P) 3, P7, P14, P21, and P30) (**Figure** **1**A). The results showed that *Serpine2* was expressed at both the transcriptional (Figure 1B) and translational (Figure 1C) levels at all stages of cochlear development after birth, and there was a significant downward trend of expression as mice aged, which was consistent with the stemness of cochlear progenitors. Therefore, we hypothesized that *Serpine2* has certain functions during cochlear development in mice and plays specific roles in cochlear progenitors.

To further explore whether *Serpine2* exerts certain effects on HC regeneration in vivo, *Serpine2* ^loxp/+^ mice were created (Figure 1D). The genotyping results of these mice are shown in Figure 1E. P1 *Serpine2* cOE mice were administrated with tamoxifen via intraperitoneal (i.p.) injection to confirm the overexpression of *Serpine2* in the basilar membrane (BM) (Figure 1F). According to the results of RT‐qPCR, significantly higher expression level of *Serpine2* was observed in *Serpine2* cOE mice compared with control mice, and the expression level of *Serpine2* showed a dose‐dependent increase in the transgenic mice (Figure 1G). Moreover, the staining of the HA‐tag in Lgr5+ progenitors indicated that *Serpine2* was successfully overexpressed in Lgr5+ progenitors (Figure 1H).

### 2.2 Ectopic IHCs Were Increased in Heterozygous *Serpine2* cOE Mice

**Figure 2:**
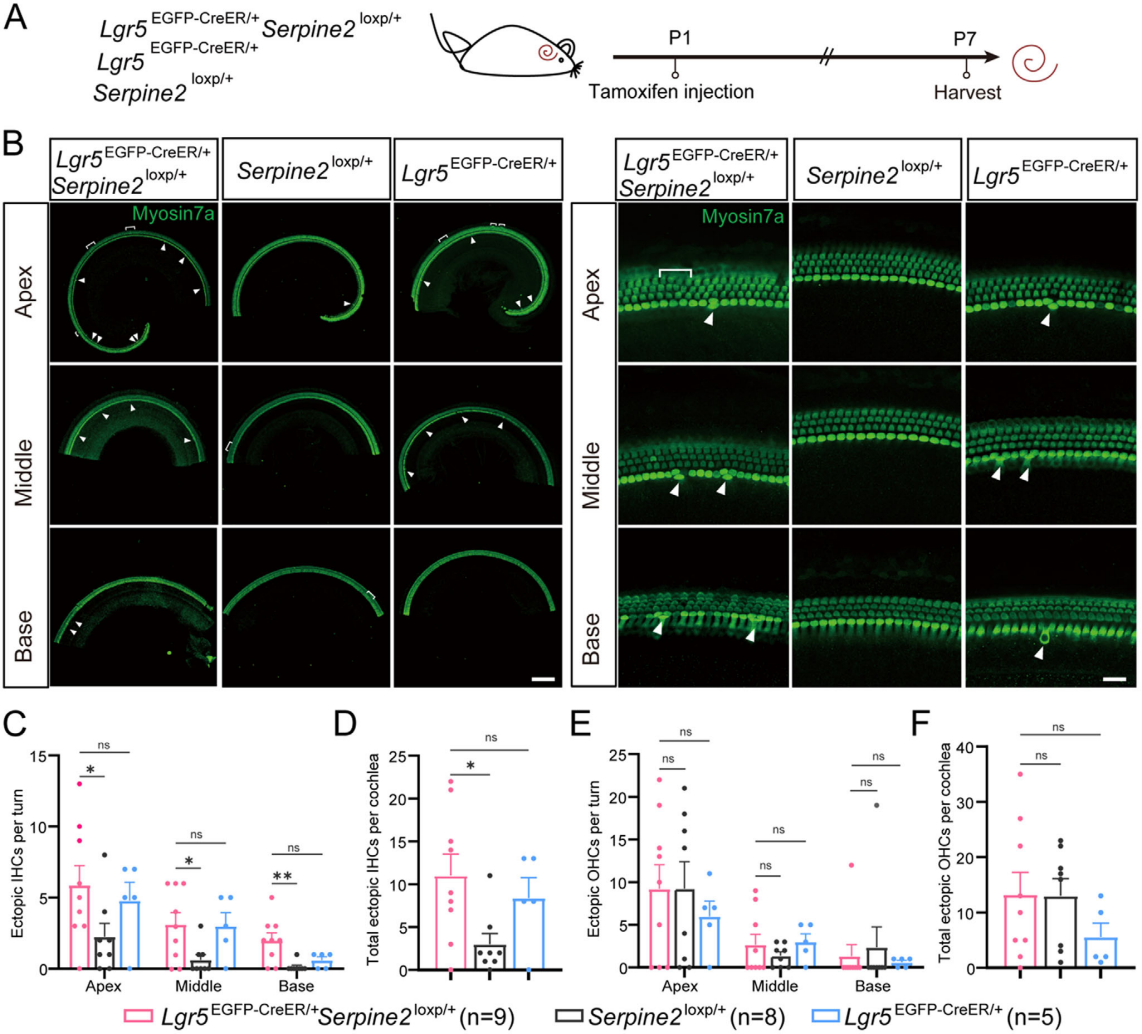
Increased ectopic IHCs in heterozygous *Serpine2* cOE mice. A) Flow chart of tamoxifen i.p. injection (0.075 mg g^−1^ body weight) and cochlear imaging and quantification at P7. B) Ectopic IHCs (indicated by arrows) and OHCs (indicated by square brackets) were observed in heterozygous *Serpine2* cOE mouse cochleae. *Lgr5* ^EGFP‐CreER/+^ mice and *Serpine2* ^loxp/+^ mice were used as control groups. Myosin7a was used as the HC marker. Scale bars on the left and right sides in (B) are 200 and 20 µm, respectively. C–F) Quantification of the ectopic IHCs in each turn (C), total ectopic IHCs (D), ectopic OHCs in each turn (E), and total ectopic OHCs (F). “*n*” refers to the number of mice used. *p < 0.05, **p < 0.01, n.s., not significant.

To explore the effect of *Serpine2* on HC regeneration, P1 heterozygous *Serpine2* cOE mice (*Lgr5* ^EGFP‐CreER/+^ *Serpine2* ^loxp/+^ mice) were administrated with tamoxifen via i.p. injection to activate the Cre enzyme and thereby overexpress *Serpine2*. The mouse cochlea was dissected at P7 for imaging (**Figure** **2**A). Both *Lgr5* ^EGFP‐CreER/+^ mice and *Serpine2* ^loxp/+^ mice were control groups. Numerous ectopic HCs were observed in heterozygous *Serpine2* cOE cochleae (Figure 2B). Statistical analysis indicated that the total ectopic IHCs number in cochlea of heterozygous *Serpine2* cOE mice was more than threefold of that of *Serpine2* ^loxp/+^ control mice, but these were not significantly different in comparison with *Lgr5* ^EGFP‐CreER/+^ control mice (Figure 2C,D). The number of ectopic IHCs showed an increasing trend from the basal turn to apical turn in heterozygous *Serpine2* cOE mice. The number of ectopic IHCs in three cochlear turns of heterozygous *Serpine2* cOE mice was more than or equal to twofold, fourfold, and 16‐fold of *Serpine2* ^loxp/+^ mice, respectively (Figure 2B,C). However, the increase in ectopic outer HCs (OHCs) in heterozygous *Serpine2* cOE mice was not significantly different compared to both control mice (Figure 2E,F). Considering that *Serpine2* is haploid overexpressed in heterozygous *Serpine2* cOE mice, which may impact the effect of *Serpine2* on promoting HC regeneration, we used homozygous *Serpine2* cOE mice for further study. In addition, different effects of haploid and diploid overexpression of *Serpine2* on HC regeneration will also be investigated.

### 2.3 Ectopic HCs Were Significantly Increased in Homozygous *Serpine2* cOE Mice

**Figure 3:**
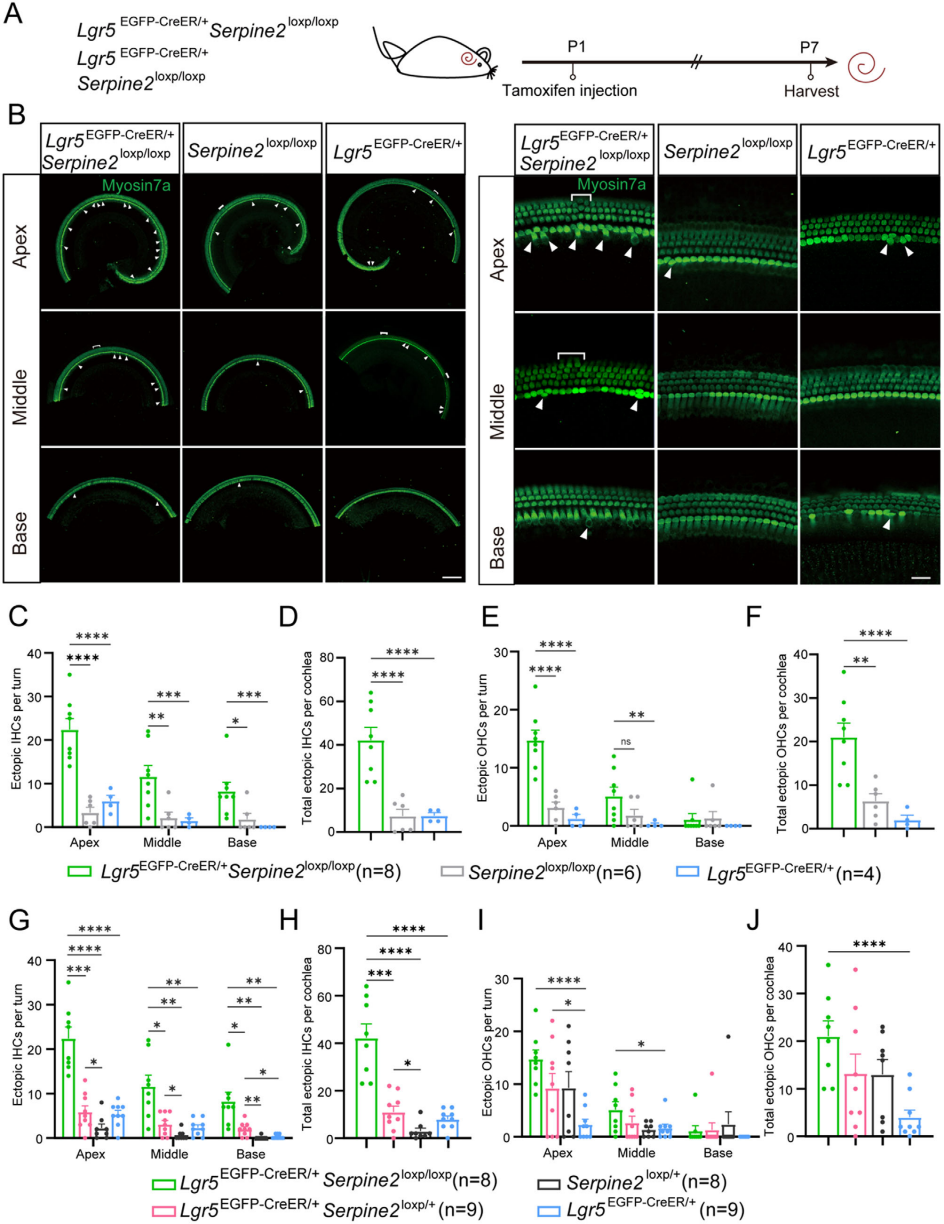
Significantly increased ectopic HCs in homozygous *Serpine2* cOE mice. A) Flow chart of tamoxifen i.p. injection (0.075 mg g^−1^ body weight) and cochlear imaging and quantification at P7. B) Ectopic IHCs (arrows) and OHCs (square brackets) were observed in homozygous *Serpine2* cOE cochleae. *Lgr5* ^EGFP‐CreER/+^ mice and *Serpine2* ^loxp/loxp^ mice were used as control groups. Myosin7a was used as the HC marker. Scale bars on the left and right sides in (B) are 200 and 20 µm, respectively. C–F) The quantification of ectopic IHCs in each turn (C), total ectopic IHCs (D), ectopic OHCs in each turn (E), and total ectopic OHCs (F). G–J) Quantification of the ectopic IHCs per turn (G), the total ectopic IHCs per cochlea (H), and quantification of the ectopic OHCs per turn (I), the total ectopic OHCs per cochlea (J) of *Lgr5* ^EGFP‐CreER/+^ mice, *Serpine2* ^loxp/+^ mice, *Lgr5* ^EGFP‐CreER/+^ *Serpine2* ^loxp/+^ mice, and *Lgr5* ^EGFP‐CreER/+^ *Serpine2* ^loxp/loxp^ mice (G). “*n*” refers to the number of mice in the experiment. *p < 0.05, **p < 0.01, ***p < 0.001, ****p < 0.0001. n.s., not significant.

To further confirm the effect of *Serpine2* on HC regeneration (**Figure** **3**A), *Serpine2* ^loxp/loxp^ were crossed with heterozygous *Serpine2* cOE mice to obtain homozygous *Serpine2* cOE mice (*Lgr5* ^EGFP‐CreER/+^ *Serpine2* ^loxp/loxp^ mice) (Figure S1A, Supporting Information). Compared to *Lgr5* ^EGFP‐CreER/+^ and *Serpine2* ^loxp/loxp^ control groups, there was a significant more than fivefold increase in ectopic IHCs of homozygous *Serpine2* cOE mice (Figure 3B,C,D). We also observed significantly more ectopic OHCs in homozygous *Serpine2* cOE mice compared to both control groups, with at least threefold increase in total ectopic OHCs (Figure 3B,E,F). Furthermore, the number of ectopic IHCs in the apical, middle, and basal turns has an increasing trend in *Serpine2* ^loxp/+^ mice, *Lgr5* ^EGFP‐CreER/+^ *Serpine2* ^loxp/+^ heterozygous cOE mice, and *Lgr5* ^EGFP‐CreER/+^ *Serpine2* ^loxp/loxp^ homozygous cOE mice. The number of ectopic OHCs in the apical turns also has an increasing trend in *Lgr5* ^EGFP‐CreER/+^ mice, *Lgr5* ^EGFP‐CreER/+^ *Serpine2* ^loxp/+^ mice, and *Lgr5* ^EGFP‐CreER/+^ *Serpine2* ^loxp/loxp^ mice (Figure 3G–J and Table S1 (Supporting Information)). These results showed that the number of ectopic OHCs and IHCs increased along with the increase of *Serpine2* expression level, demonstrating a dose‐dependent effect of *Serpine2* on ectopic HC number. Therefore, we concluded that overexpression of *Serpine2* in Lgr5+ progenitors can significantly induce HC regeneration with a dose‐dependent effect in the cochlea of neonatal mice.

### 2.4 Proliferation of Lgr5+ Progenitors Was Not Observed in *Serpine2* cOE Mice

**Figure 4:**
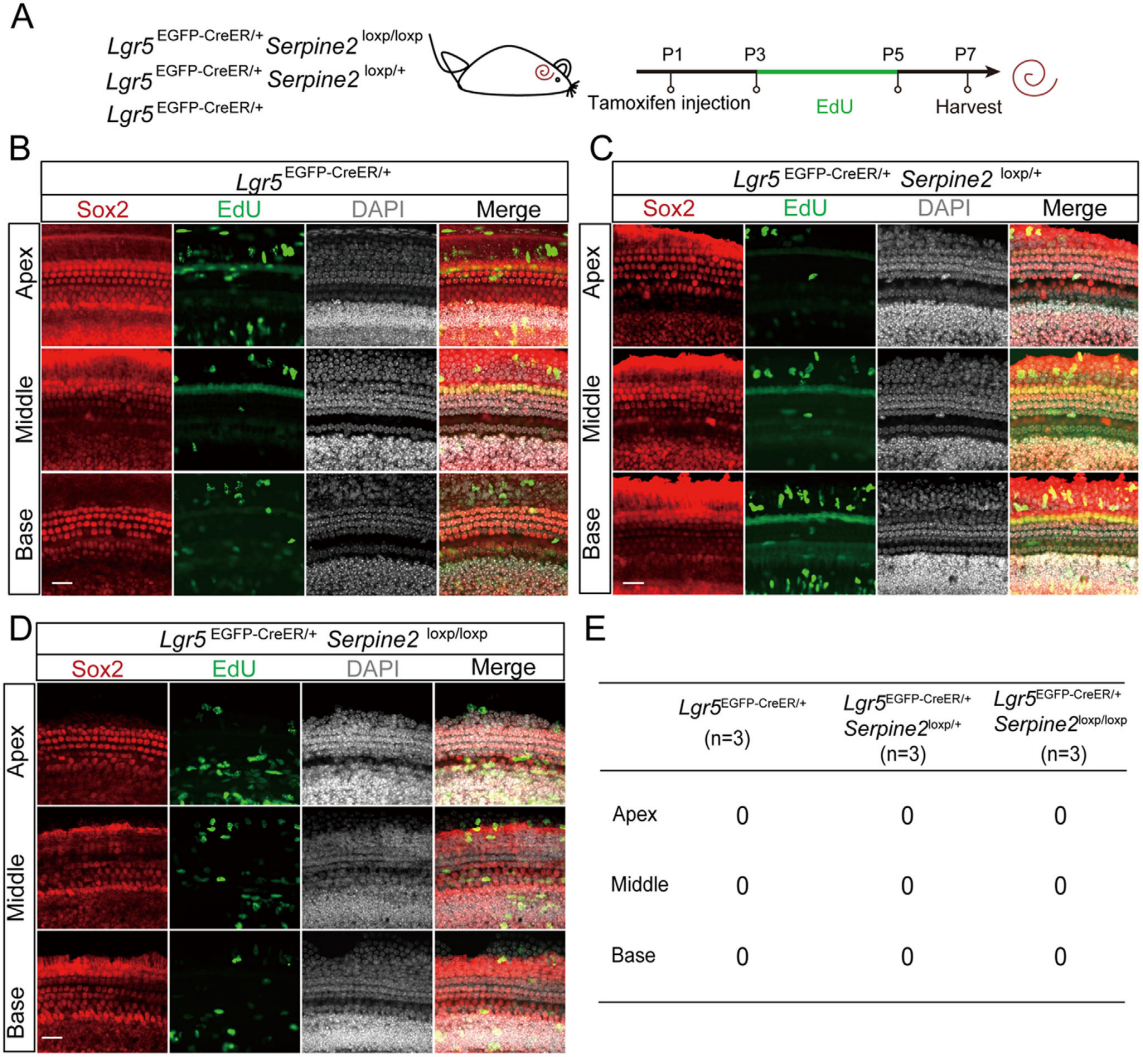
EdU assay in the cochleae of *Serpine2* cOE mice. A) Flow chart of the EdU assay. EdU (0.05 mg g^−1^ body weight) was i.p. injected from P3 to P5 to label proliferating cells. B–D) EdU was stained (green) in the cochleae of *Lgr5* ^EGFP‐CreER/+^ mice (B), heterozygous *Serpine2* cOE mice (C), and homozygous *Serpine2* cOE mice (D). Sox2 (red) was used as the SC marker, and DAPI (white) was used to label the nucleus. Scale bars are 20 µm in (B–D). E) Quantification of EdU+ SCs in *Serpine2* cOE cochleae. “*n*” refers to the number of mice used.

Previous studies have demonstrated that HC regeneration can be induced through mitotic regeneration, direct transdifferentiation, or both.^[^ ^12^ ^]^ Thus, we probed the underlying mechanism of *Serpine2* cOE induced HC regeneration in vivo. To determine if HCs could be regenerated through mitosis, heterozygous and homozygous *Serpine2* cOE mice were administrated with tamoxifen at P1 and with EdU from P3 to P5 both via i.p. injection to label proliferating cells. *Lgr5* ^EGFP‐CreER/+^ mice were used as control group and were treated the same way (**Figure** **4**A). Staining and statistical analysis showed that neither *Serpine2* cOE mice nor control mice contained any EdU+ SCs (Figure 4B–E), suggesting that the ectopic HCs may not be regenerated through mitotic generation.

### 2.5 *Serpine2* cOE Promotes HC Regeneration by Inducing the Direct Transdifferentiation of Lgr5+ Progenitors

**Figure 5:**
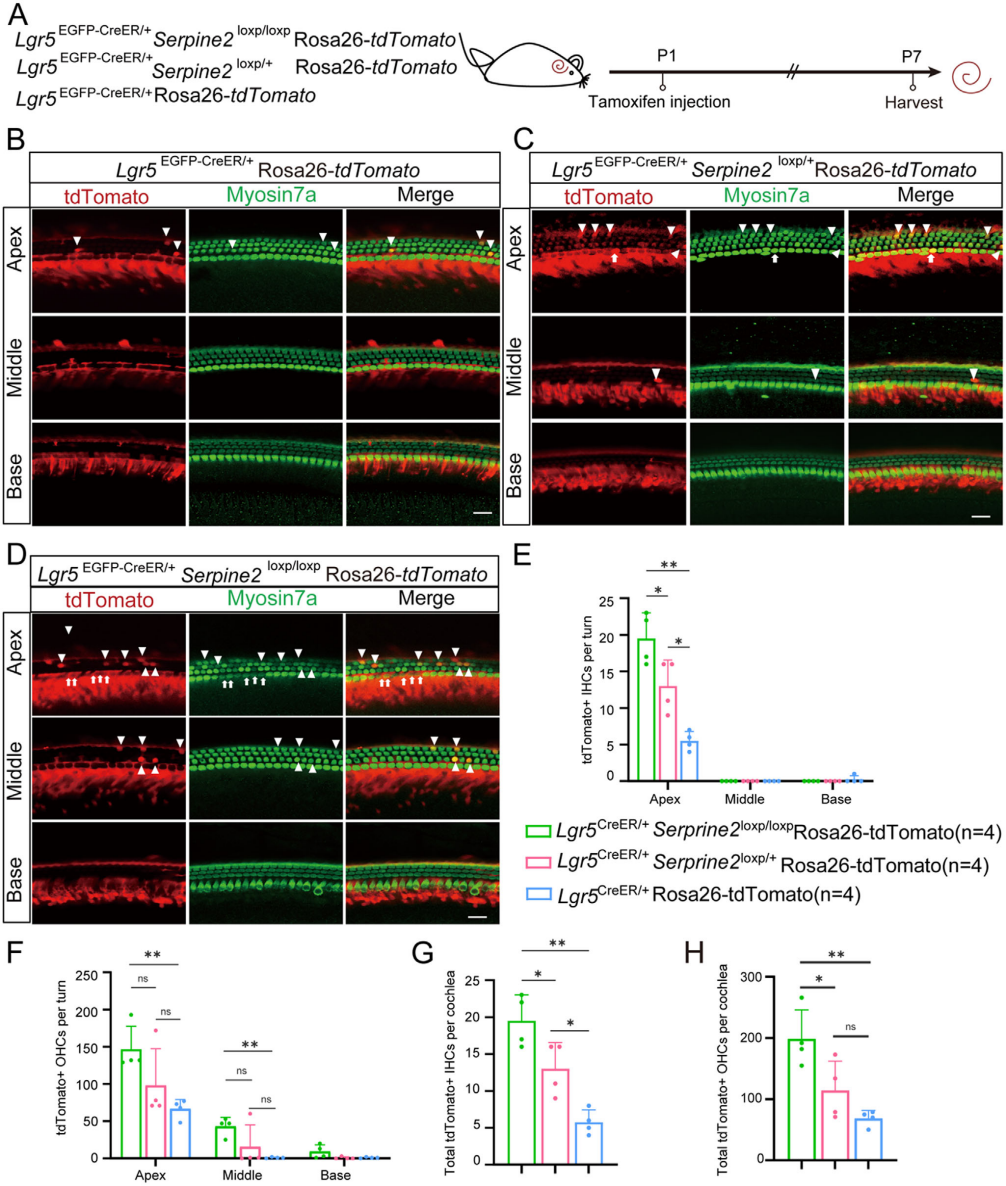
Lineage tracing of Lgr5+ progenitors in *Serpine2* cOE mice. A) Flow chart of the lineage tracing assay. B–D) Lineage tracing images of *Lgr5* ^EGFP‐CreER/+^Rosa26‐*tdTomato* mice (B), *Lgr5* ^EGFP‐CreER/+^ *Serpine2* ^loxp/+^Rosa26‐*tdTomato* mice (C), and *Lgr5* ^EGFP‐CreER/+^ *Serpine2* ^loxp/loxp^Rosa26‐*tdTomato* mice (D). tdTomato+ IHCs and tdTomato+ OHCs are indicated by arrows and arrowheads, respectively. Scale bars are 20 µm in (B–D). E–H) Quantification of tdTomato+ IHCs (E) and OHCs (F) in each turn and the total number of tdTomato+ IHCs (G) and OHCs (H) per cochlea. “*n*” refers to the number of mice used. *p < 0.05, **p < 0.01, n.s., not significant.

Based on the above results, we obtained two kinds of triple‐positive mice, *Lgr5* ^EGFP‐CreER/+^ *Serpine2* ^loxp/+^Rosa26‐*tdTomato* and *Lgr5* ^EGFP‐CreER/+^ *Serpine2* ^loxp/loxp^Rosa26‐*tdTomato* for lineage tracing (Figure S1B, Supporting Information). The *Lgr5* ^EGFP‐CreER/+^Rosa26‐*tdTomato* mice were used as the control group. Tamoxifen was i.p. injected into P1 mice to active Cre enzyme, and the Myosin7a and double‐positive HCs were observed and quantified at P7 (**Figure** **5**A). Compared to the control group, both heterozygous and homozygous triple‐positive mice cochlea contained significantly more tdTomato+ IHCs in their cochlear apical turns, with an increase of at least twofold and threefold, respectively. In particular, significantly more tdTomato+ OHCs were observed in the apical and middle turns of the cochlea in homozygous triple‐positive mice compared with the control group (Figure 5B–H and Figure S2A–C (Supporting Information)). Taken together, the results of the EdU assay and lineage tracing assay suggested that *Serpine2* cOE in cochlear Lgr5+ progenitors in vivo promotes HC regeneration and results in a significant increase in ectopic HCs, especially IHCs, and that this is likely by triggering Lgr5+ progenitors to transdifferentiate directly into HCs instead of inducing mitotic regeneration. To evaluate the effect of *Serpine2* overexpression on mice hearing ability, we performed auditory brainstem response testing on P30 *Serpine2* cOE mice and control mice, respectively, and the results showed that *Serpine2* overexpression had no effect on mice hearing ability compared to the control mice (Figure S3A–C, Supporting Information).

### 2.6 *Serpine2* Knockdown ex vivo and in vivo Can Both Inhibit Neonatal HC Regeneration

**Figure 6:**
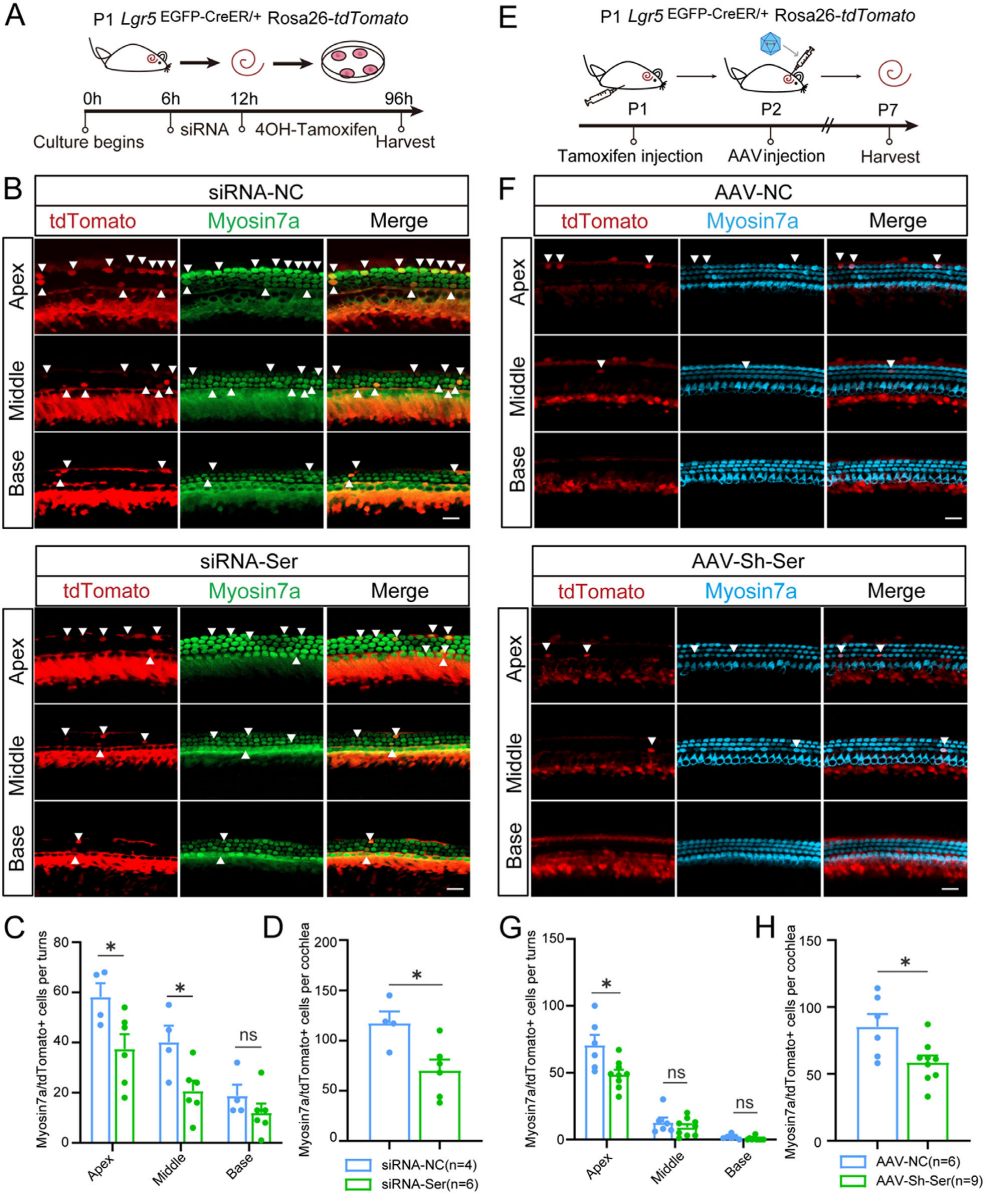
Lineage tracing of Lgr5+ progenitors after *Serpine2* knockdown ex vivo and in vivo. A) Flow chart of the lineage tracing assay after *Serpine2* knockdown by siRNA ex vivo. B) Lineage tracing images of *Lgr5* ^EGFP‐CreER/+^Rosa26‐*tdTomato* mice treated with siRNA‐NC and siRNA‐Ser. Arrows indicate tdTomato+ HCs. C,D) Quantification of tdTomato+ HCs in each turn (C) and the total number of tdTomato+ HCs per cochlea (D) after *Serpine2* knockdown ex vivo. E) Flow chart of the lineage tracing assay after *Serpine2* knockdown by AAV in vivo. F) Lineage tracing images of *Lgr5* ^EGFP‐CreER/+^Rosa26‐*tdTomato* mice injected with AAV‐Sh‐Ser and AAV‐NC. Arrows indicate tdTomato+ HCs. Scale bars are 20 µm in (B) and (F). G,H) Quantification of tdTomato+ HCs in each turn (G) and the total number of tdTomato+ HCs per cochlea after *Serpine2* knockdown in vivo (H). *p < 0.05, n.s., not significant.

To further prove the effect of *Serpine2* on HC regeneration, we then knockdown *Serpine2* ex vivo and in vivo by using siRNA and Adeno‐associated virus (AAV), respectively, in the cochlea of *Lgr5* ^EGFP‐CreER/+^Rosa26‐*tdTomato* mice for lineage tracing. We first screened the siRNA with the best knockdown efficiency in HEI‐OC1 cell line (Figure S4A,B, Supporting Information). And then the most efficient siRNA‐*Serpine2*‐1243 (referred to as siRNA‐Ser hereafter) was transfected into cochlear explants of P1 *Lgr5* ^EGFP‐CreER/+^Rosa26‐*tdTomato* mice to knockdown *Serpine2* ex vivo, and tdTomato+ HCs were quantified after four days of culture (**Figure** **6**A). The knockdown efficiency of siRNA‐*Serpine2*‐1243 on cochlear tissue was verified at the protein level (Figure S4C,D, Supporting Information). Compared to the control group, *Serpine2* knockdown can significantly reduce the number of tdTomato+ HCs in apical and middle turns of cochlear explants, with at least 1.5‐fold and 1.9‐fold, respectively (Figure 6B–D). We then constructed pAAV2‐shRNA plasmid (Figure S4E, Supporting Information) by using siRNA‐Ser sequence and packaged it into the AAV‐inner ear (AAV‐ie) (referred to as AAV‐Sh‐Ser hereafter) to investigate the effect of *Serpine2* knockdown on HC regeneration in vivo*. Lgr5* ^EGFP‐CreER/+^Rosa26‐*tdTomato* mice were injected intraperitoneally with tamoxifen at P1 and were injected with AAV‐Sh‐Ser via round window membrane (RWM) at P2. The mice were sacrificed at P7 to quantify tdTomato+ HCs (Figure 6E). The knockdown efficiency of AAV‐Sh‐Ser in vivo was verified at the protein level (Figure S4F,G, Supporting Information). We also verified the transduction efficiency of AAV‐Sh‐Ser in SCs by using immunofluorescence imaging (Figure S4H,I, Supporting Information). Similar to the ex vivo results, in vivo knockdown of *Serpine2* by AAV‐Sh‐Ser in neonatal mice cochlea also significantly reduced the number of tdTomato+ HCs in apical turn (Figure 6F–H). Therefore, our ex vivo and in vivo results both indicated that knockdown of *Serpine2* can significantly reduce HC regeneration in neonatal mice cochlea, which supports and is consistent with our *Serpine2* cOE mice results.

### 2.7 Single‐Nucleus Transcriptomic Analysis of *Serpine2* cOE Mice Cochlea

**Figure 7:**
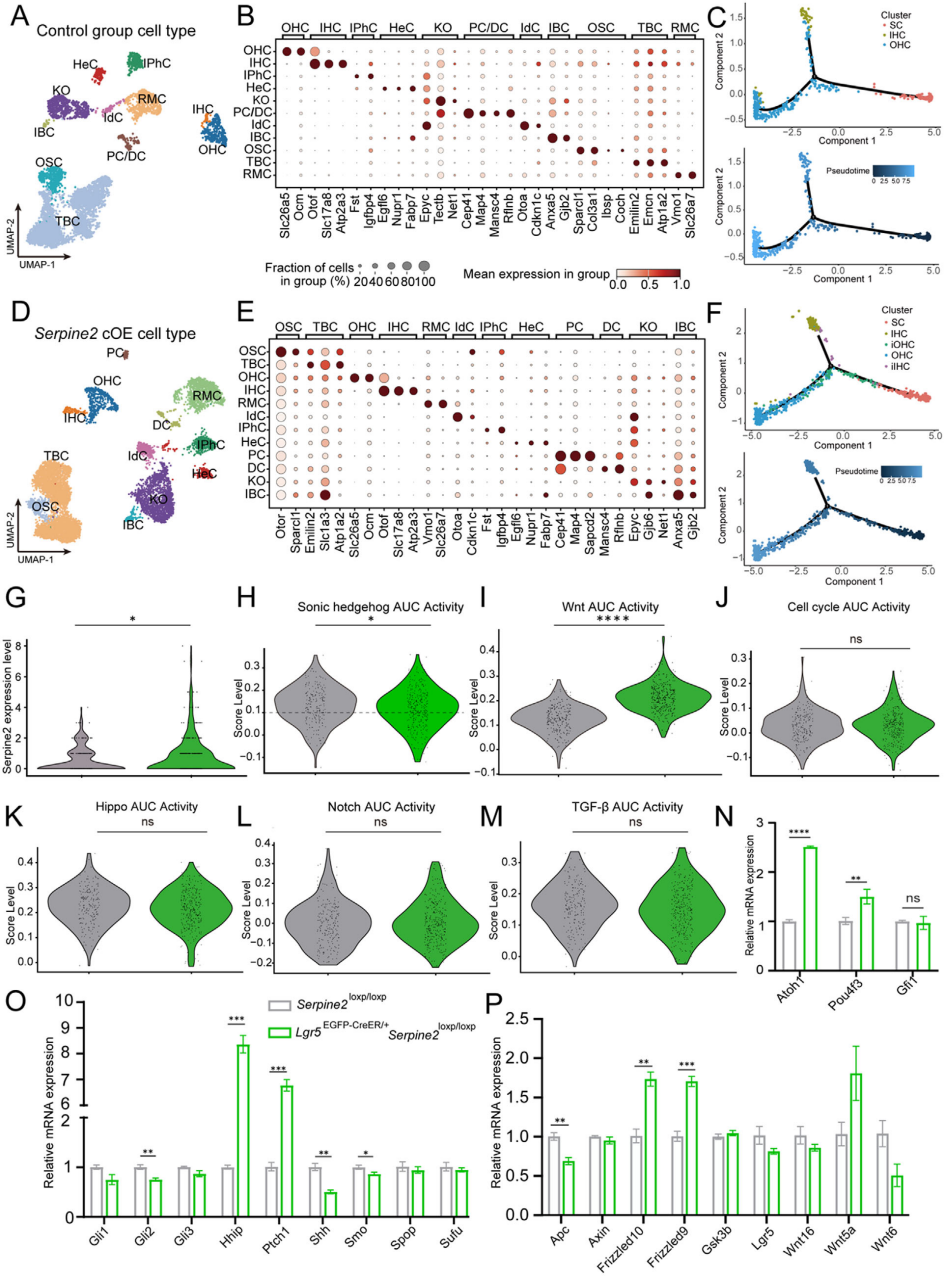
Single‐nucleus transcriptomic analysis of *Serpine2* cOE mice cochlea. A,B) UMAP plot (A) and Dotplot (B) show 11 distinct cell types in the control *Serpine2* ^loxp/loxp^ mouse cochleae. C) The trajectory manifold of HCs from SCs of *Serpine2* ^loxp/loxp^ mouse using the Monocle 2 R package. D,E) UMAP plot (D) and Dotplot (E) show 12 distinct cell types in the *Lgr5* ^CreER/+^ *Serpine2* ^loxp/loxp^ mouse cochleae. F) The trajectory manifold of HCs from SCs of *Lgr5* ^CreER/+^ *Serpine2* ^loxp/loxp^ mouse using the Monocle 2 R package. G) *Serpine2* expression level in snRNA‐seq data of *Serpine2* cOE mice cochlea and control mice cochlea. H–M) AUC pathway scoring of SHH (H), Wnt (I), cell cycle (J), Hippo (K), Notch (L), and TGF‐β (M) signaling pathway activity in *Serpine2* ^loxp/loxp^ and *Lgr5* ^CreER/+^ *Serpine2* ^loxp/loxp^ mouse cochleae. N–P) RT‐qPCR quantification of the expression of transcription factors (N), SHH signaling pathway genes (O), and Wnt signaling pathway genes (P) in the BM of P7 *Serpine2* ^loxp/loxp^ and *Lgr5* ^CreER/+^ *Serpine2* ^loxp/loxp^ mouse cochleae. IPhC, inner phalangeal cell; IBC, inner border cell; IHC, inner hair cell; OHC, outer hair cell; KO, Kölliker's organ cell; HeC, Hensen's cell; TBC, tympanic border cell; IdC, interdental cell; DC, Deiters’ cell; OSC, outer sulcus cell; RMC, Reissner's membrane cell; PC, pillar cell; SC, supporting cell; iOHC, immature outer hair cell; iIHC, immature inner hair cell. *n* = 4, *p < 0.05, **p < 0.01, ***p < 0.001, ****p < 0.0001, n.s., not significant.

To further explore the mechanism of *Serpine2* in HC regeneration, we performed snRNA‐seq to characterize the transcriptome difference at single cell level. We succeeded in capturing 5846 and 7725 single nuclei from the control group (*Serpine2* ^loxp/loxp^) and *Serpine2* cOE group (*Lgr5* ^EGFP‐CreER/+^ *Serpine2* ^loxp/loxp^) cochleae, respectively. Based on the expression of known markers, we identified distinct cell types in both groups, including OHCs (*Slc26a5*), IHCs (*Slc17a8*, Atp2a3, and *Otof*), and different kinds of SCs (**Figure** **7**A,B,D,E). The percentage of HCs in the control group and *Serpine2* cOE group is 5.87% and 7.21%, respectively (Table S5, Supporting Information), which is consistent with and further supports our phenotype results that *Serpine2* cOE results in increased number of HCs. We defined a HC subpopulation with high expression level of *Lgr5* and some SCs markers as immature HCs (iHCs) which are thought to represent HCs in the process of transdifferentiation or newly generated HC subtypes. We then employed Monocl2 trajectory analysis to visualize the differentiation trajectory and trend SCs in two groups. The analysis revealed branched trajectories in both groups, with a part subset of immature IHCs (iIHCs) and immature OHCs (iOHCs) at the middle of the IHC and OHC development trajectory in the *Serpine2* cOE group (Figure 7C,F), which is consistent with and further supports our results that *Serpine2* cOE induces HC regeneration.

To study the genes and pathways regulated by *Serpine2* in HC regeneration, we targeted Lgr5+ cells in specific cell populations (HC, Deiters’ cell (DC), pillar cell (PC), Hensen's cell (HeC), inner border cell (IBC)) from both the control and *Serpine2* cOE groups to perform further gene expression and signaling pathway analysis. We noticed that *Serpine2* expression in these Lgr5+ cells is indeed significantly higher in *Serpine2* cOE group compared with the control group (Figure 7G). We first performed differentially expressed genes (DEGs) ranking for Lgr5+ cells in both groups, and conducted Metascape enrichment analysis of DEGs (log2FC > 0, *p*‐value < 0.05) in the target Lgr5+ cell populations of *Serpine2* cOE group, which showed that these DEGs are involved in the regulation of pathways involved in differentiation and development, such as cardiac muscle cell differentiation, positive regulation of developmental growth, and Wnt signaling pathways (Figure S5, Supporting Information). Considering that there are many pathways reported to regulate HC regeneration, we performed regulon activity (AUC) scoring to assess the activity of these relevant pathways such as SHH, Wnt, cell cycle, Hippo, Notch, and TGF‐β signaling pathway activity in Lgr5+ cell subpopulations of both groups (Figure 7H–M). The results revealed that the *Serpine2* cOE group a lower score in SHH pathway and a higher score in Wnt pathway compared with the control group, while there were no significant differences in other pathways. We then performed RT‐qPCR to further verify our sequencing results and found that in the SHH signaling pathway, *Hhip* and *Ptch1* expression levels were upregulated, while *Smo*, *Shh*, and *Gli2* expression levels were downregulated in the *Serpine2* cOE group, which was consistent with the sequencing data that SHH pathway is downregulated (Figure 7O). However, RT‐qPCR results showed no significant gene expression difference in Wnt pathway (Figure 7P). Additionally, we also tested the expression of key transcription factors *Atoh1*, *Pou4f3*, and *Gfi1* which are reported to play important roles in HC differentiation. We found that *Atoh1* and *Pou4f3* exhibited significantly higher mRNA levels in *Serpine2* cOE group (Figure 7N). These results suggested that *Serpine2* cOE induce HC regeneration mainly through inhibiting SHH pathway and inducing *Atoh1* and *Pou4f3* transcription factors.

## 3 Discussion

HCs can be stimulated by mechanical sound waves and transmit these sound signals to the cochlear spiral ganglion neurons in the form of electrical signals.^[^ ^3a^ ^]^ However, HCs are vulnerable to noise, ototoxic drugs, aging, and virus infection, and loss of HCs eventually leads to SNHL.^[^ ^2b^ ^]^ Lgr5+ progenitors in neonatal mice have limited capacity for HC regeneration through direct transdifferentiation and/or mitotic regeneration,^[^ ^2^, ^31^ ^]^ and how to further promote HC regeneration from Lgr5+ progenitors in order to replenish lost HCs is one of the main focuses of research into SNHL. Although the proliferation and differentiation of Lgr5+ progenitors could be regulated by multiple genes and signaling pathways, the regenerated HCs are neither abundant enough nor mature enough to restore normal hearing function.^[^ ^21^, ^32^ ^]^ We previously screened several novel genes, including *Serpine2*, that may be involved in regulating the proliferation and/or differentiation of Lgr5+ progenitors by performing the sphere‐forming and differentiation assays in vitro (unpublished data).

In this study, we comprehensively studied the function of *Serpine2* in HC regeneration in vivo. We first tested the expression pattern of *Serpine2* and found that *Serpine2* is expressed in mouse cochleae at all stages after birth, and its expression level declines as the mice age. Considering that its downward expression trend is consistent with the stemness of Lgr5+ progenitors in the mouse cochlea,^[^ ^33^ ^]^ *Serpine2* may be important for cochlear development.

In our study, both heterozygous and homozygous conditional *Serpine2* overexpression in Lgr5+ progenitors in mouse inner ear resulted in increased numbers of ectopic HCs, especially IHCs. Ectopic OHCs were mostly found in the apical and middle turns in homozygous overexpression mice, while ectopic IHCs were observed in all three cochlear turns, indicating that *Serpine2* can promote HC regeneration in the cochlea to a certain extent. Studies have shown that Lgr5+ progenitors in the apical turn have stronger proliferation and regeneration capabilities compared to those in the basal turn, and numerous genes are involved in this difference in ability,^[^ ^24b^ ^]^ which is consistent with our results.

Interestingly, we found that overexpression of *Serpine2* in Lgr5+ progenitors induced ectopic HCs in a dose‐dependent manner, and heterozygous *Serpine2* overexpression resulted in a smaller increase only in ectopic IHCs, while homozygous *Serpine2* overexpression led to significantly greater increases in both IHCs and OHCs. This phenomenon may be due to the difference of *Serpine2* expression level in the cochlea of heterozygous and homozygous overexpression mice, which further suggests that *Serpine2* plays an important role in HC regeneration. At the same time, we found that *Serpine2* knockdown inhibits HC regeneration in neonatal mice cochlea both ex vivo and in vivo, which further prove that *Serpine2* plays a crucial role in HC regeneration during the neonatal period.

As previously reported, HCs can be regenerated via mitotic regeneration and/or direct transdifferentiation. The former involves SCs or progenitors reentering the cell cycle and undergoing mitosis and subsequently differentiating into HCs, whereas the latter refers to the direct differentiation of SCs or progenitors into HCs without first undergoing mitosis.^[^ ^3^, ^34^ ^]^ To identify the HC regeneration pathways induced by overexpression of *Serpine2*, we performed EdU and lineage tracing assays. Significantly more tdTomato+ HCs were seen in *Serpine2* cOE mice than in control mice. By contrast, cochlea of neither *Serpine2* cOE mice nor control mice contained EdU+ SCs, indicating ectopic HCs induced by *Serpine2* cOE are likely regenerated in vivo via direct transdifferentiation of Lgr5+ progenitors and not through mitotic regeneration. Whether *Serpine2* can induce the proliferation of Lgr5+ progenitors in vitro or under other circumstances needs further study.

Based on our snRNA‐seq analysis, *Serpine2* cOE group contains more HCs than control group, and has some newly regenerated HCs we named iHCs that are probably HCs transdifferentiating from Lgr5+ progenitors, which support our results that *Serpine2* induces HC regeneration. Based on the mRNA validation results, we found that *Serpine2* cOE suppresses SHH signaling activity, as evidenced by decreased mRNA levels of the activator *Gli2*, the *Shh* receptor, and its downstream effector *Smo*, along with increased mRNA levels of the negative regulator *Hhip* and the inhibitory receptor *Ptch1*. Both *Ptch1* and *Gli2* are critical for HC differentiation and vascular striae development.^[^ ^35^ ^]^ It was reported that high SHH signaling/*Gli2* facilitates the preservation of progenitor cell identity in the cochlea, whereas low SHH signaling/*Gli2* promotes HC differentiation.^[^ ^35^ ^]^ Research has also shown that the absence of *Serpine2* could cause increased expression of *Gli1* and *Ptch1* during mouse brain development, indicating that *Serpine2* acts as a negative modulator of SHH signal transduction.^[^ ^36^ ^]^ These reports support and are consistent with our findings that *Serpine2* is likely to induce HC regeneration by downregulating the SHH pathway. Many important transcription factors are reported to be involved in HC differentiation and regeneration. Among them, *Atoh1* is necessary and sufficient for HC formation, and *Pou4f3* could cooperate with *Atoh1* to regulate HC differentiation.^[^ ^21^ ^]^ Moreover, the mRNA expression levels of *Atoh1* and *Pou4f3* were significantly elevated in the *Serpine2* cOE group, indicating that *Serpine2* may work synergistically with these transcription factors to regulate HC regeneration. Therefore, we speculate that *Serpine2* is likely to induce HC regeneration through inhibiting SHH signaling pathways and inducing *Atoh1* and *Pou4f3* transcription factors, which needs further careful study to confirm the detailed regulation mechanisms in the future.

Moreover, it has been demonstrated that a large number of genes and signaling pathways regulate the differentiation and cell density of inner ear progenitors, such as *Atoh1*, *Pou4f3*, *Gfi1*, *Foxg1*, Hippo, Notch, and others,^[^ ^17^, ^23^, ^37^ ^]^ and that coregulation of two or three genes and signaling pathways can further promote HC regeneration.^[^ ^15^, ^21^, ^38^ ^]^ Therefore, it is also meaningful to determine if other factors or signaling pathways can cooperate with *Serpine2* to promote HC regeneration further and to explore the most effective strategy of multigene cooperative regulation to achieve sufficient functional HC regeneration in vivo in neonatal and even in adult mammals. AAV is found to be an efficient and safe gene delivery tool used widely in gene therapy in many diseases and tissues.^[^ ^3c^ ^]^ In the inner ear, there are also several reports that AAV can be used to restore hearing of patients with hereditary deafness in clinic.^[^ ^39^ ^]^ AAV‐ie and AAV‐ie‐K558R were recently reported to be a good gene delivery tool for cochlear HCs and SCs with high efficiency, with the capacity to rectify inherited hearing impairment and HC regeneration.^[^ ^3^, ^32^, ^37^ ^]^ Combining *Serpine2* with signaling pathways or transcription factors that induce HC regeneration, such as the SHH signaling pathway, *Atoh1*, and *Pou4f3*, AAV‐mediated SC and progenitor reprogramming to regulate HC regeneration has great clinical potential. Furthermore, inner ear organoids represent a valuable tool for accelerating drug discovery in the field of inner ear research and for identifying potential therapeutic targets for hearing loss.^[^ ^40^ ^]^ In the future, these organoids could be employed to investigate optimal strategies for gene coregulation aimed at inducing HC regeneration, thereby expediting the research process and testing the clinical applicability of these findings.

In conclusion, we conditionally overexpressed *Serpine2* in Lgr5+ progenitors in the newborn mouse cochlea by using *Serpine2* cOE mice, and observed significantly more ectopic HCs compared with control mice. Conversely, *Serpine2* knockdown inhibits HC regeneration in neonatal mice cochlea both ex vivo and in vivo. Moreover, the results of the EdU assay, lineage tracing assay, and trajectory analysis suggest that ectopic HCs are likely regenerated by direct transdifferentiation rather than mitotic regeneration. Currently, our results suggest that *Serpine2* may promote HC regeneration via inhibiting the SHH signaling pathway and combining with *Atoh1* and *Pou4f3*. However, the underlying mechanism between *Serpine2* and SHH signaling requires further investigation.

## 4 Experimental Section

### Animals

*Lgr5* ^EGFP‐IRES‐CreERT2^ mice (Stock number, 008875) and Rosa26‐*tdTomato* reporter mice (Stock number, 007914) were purchased from the Jackson Laboratory.^[^ ^12^ ^]^ BIOCYTOGEN (Beijing, China) constructed the *Serpine2* ^loxp/+^ (C57BL/6JHipp11‐loxp‐STOP‐loxp‐*Serpine2*) transgenic mice. Briefly, CRISPR/Cas9 technology was used to inset a targeting vector into the Hipp11 locus of C57BL/6 J mice. The target vector consisted of the exogenous *Serpine2* gene, a stop element flanked by two loxp sequences, and a 3 × HA tag fragment. All of the animal experiments were conducted according to protocols permitted by the Southeast University Animal Care and Use Committee (experiment number: 20210302031), which were aligned with the National Institutes of Health Guidelines for the Care and Use of Laboratory Animals. The best was done to alleviate animal suffering and to decrease the quantity of animals used.

### Genotyping of Mice

Mouse tail tips were used to extract DNA by lysis with 50 mm NaOH at 98 °C for 1 h, followed by termination with 1 m Tris‐HCl pH 8.0 (Solarbio, T1150). The genotyping PCR system was composed of 5 µL PCR mix (Vazyme, p131‐02), 1.5 µL genomic DNA, 1 µL primers, and 2.5 µL H_2_O. PCR conditions were an initial denaturing phase of 3 min at 94 °C, followed by 38 cycles of 30 s denaturation at 94 °C, 45 s annealing at 58 °C, and 45 s extension at 72 °C. The primers used were shown in Table S2 (Supporting Information).

### Conditional Overexpression of *Serpine2* in Cochlear Lgr5+ Progenitors In Vivo

To conditionally overexpress *Serpine2* in Lgr5+ progenitors, *Lgr5* ^EGFP‐CreER/+^ mice and *Serpine2* ^loxp/loxp^ mice were crossed to get heterozygous *Serpine2* cOE mice (*Lgr5* ^EGFP‐CreER/+^ *Serpine2^l^* ^oxp/+^ mice) and homozygous *Serpine2* cOE mice (*Lgr5* ^EGFP‐CreER/+^ *Serpine2* ^loxp/loxp^ mice) (Figure S1A, Supporting Information). The control groups were *Lgr5* ^EGFP‐CreER/+^ mice, *Serpine2* ^loxp/+^ mice, and *Serpine2* ^loxp/loxp^ mice. Tamoxifen (Sigma, T5648, 0.075 mg g^−1^ body weight) was administered to both *Serpine2* cOE mice and control mice at P1 through i.p. injection as previously reported to activate the Cre enzyme.^[^ ^12^ ^]^ All mouse cochleae were dissected at P7 for further experiments.

### RNA Extraction and RT‐qPCR

Cochleae were homogenized in a high throughput tissue grinder (GallopTech, Grinder‐48) in 1 mL Trizol (Invitrogen,15596026), and RNA was extracted by adding 200 µL chloroform (Sinopharm Chemical Reagent Co., Ltd., 10006818) and precipitated with 500 µL isopropyl alcohol (Sinopharm Chemical Reagent Co., Ltd., 40064360). The total RNA was washed with 75% ethanol twice before dissolving in diethyl pyrocarbonate water. The reverse transcription kit (ThermoFisher, K1622) was used for reverse transcription to obtain cDNA. The reverse transcription PCR settings were 42 °C for 60 min and then 70 °C for 5 min. RT‐qPCR was performed by using SYBR Green (Roche, 49139140001) following the manufacturer's instructions to quantify the gene expression level. Table S3 (Supporting Information) shows the RT‐qPCR primers used. The data were normalized to the housekeeping gene *β‐actin*, and 2^‐∆∆CT^ method was applied to quantify the relative gene expression level.

### Protein Extraction and Western Blot

The cochleae from mice of different ages were homogenized in a high throughput tissue grinder in 200 µL RIPA buffer (Epizyme, PC101) supplemented with 4 µL 50 × protease inhibitor mixture (Roche, 04693132001). The protein sample was mixed with 5 × sodium dodecyl sulfate– polyacrylamide gel electrophoresis (SDS‐PAGE) loading buffer (Epizyme, LT101) and heated at 98 °C for 10 min. After electrophoresis on 10% SDS‐PAGE gel (Epizyme, PG112), the samples were transferred to a 0.45 µm PVDF membrane (Millipore, IPVH00010). The membrane was blocked with 5% v/v skim milk in 1 × 0.1% Tween‐20 in 1 × Tris‐base saline (TBST buffer) at room temperature for 2 h, and then incubated with the primary antibody of Gapdh (Abmart, M20006, 1:2000 dilution) and *Serpine2* (Abcam, Ab154591, 1:400 dilution) overnight at 4 °C. After 6 times washing with 1 × TBST buffer (5 min each time), the membranes were incubated with secondary antibodies of goat anti‐rabbit or goat anti‐mouse IgG‐HRP (Abmart, M21001; M21002, 1:2000 diluted in TBST buffer) at room temperature for 2 h. The SuperPico ECL Chemiluminescence Kit (Vazyme, E422) was used to detect the protein signals, and images were obtained by using a Tanon 2500R imaging system.

### Immunofluorescent Staining

For P7 mice, cochleae were fixed in 4% paraformaldehyde (Solarbio, P1110) at room temperature for 2 h. The cochleae were decalcified for 20 min in 0.5 m ethylenediaminetetraacetic acid after washing with phosphate buffer saline (PBS) for 3 times. The cochlear basilar membrane tissues were dissected into the apical, middle, and basal turns with sharp forceps (Dumont, 0209‐5‐PO) in cold Hank's balanced salt solution. The cochleae were attached to the slides coated with Cell‐Tak (Corning, 354240) and then blocked in blocking medium (5% donkey serum, 0.02% sodium azide, 0.5% Triton X100, and 1% bovine serum albumin in PBS, pH 7.4) at room temperature for 2 h. After incubated with primary antibodies of Myosin7a (Proteus Bioscience, 25‐6790, 1:400 dilution; DSHB, 138‐1, 1:1000 dilution) and HA (Abmart, M20003, 1:500 dilution) at 4 °C overnight, the cochleae were incubated with DAPI (Solarbio, C0060, 1:1000 dilution) and secondary antibody for 1 h and coated in antifade fluorescence mounting medium (Solarbio, S2100) on the next day. Donkey anti‐goat IgG (H+L) secondary antibody Alexa Fluor 647 conjugate (Jackson, 705‐605‐003, 1:400 dilution), donkey anti‐mouse IgG (H+L) secondary antibody Alexa Fluor 488 and 555 conjugate (Jackson, 115‐545‐003, 115‐165‐003, 1:400 dilution), and donkey anti‐rabbit IgG (H+L) secondary Antibody Alexa Fluor 488 and 555 conjugate (Jackson, 111‐545‐003, 111‐165‐003, 1:400 dilution) were secondary antibodies used in this study. The immunofluorescence images were captured with Zeiss LSM 700 and 900 confocal microscopes. The representative images, such as ectopic HCs, EdU+ SCs, and tdTomato+ HCs, were processed and optimized by using ImageJ software. For the data quantification, all the ectopic IHCs/OHCs, EdU+ SCs, and tdTomato+ HCs throughout the cochlea of each mouse were imaged and quantified, and the data were presented as per turn or per cochlea by GraphPad software.

### EdU Staining In Vivo

*Lgr5* ^EGFP‐CreER/+^ *Serpine2* ^loxp/loxp^ and *Lgr5* ^EGFP‐CreER/+^ *Serpine2* ^loxp/+^ mice were i.p. injected with Tamoxifen (0.075 mg g^−1^ body weight) at P1, and with EdU (Sigma, 900584, 0.05 mg g^−1^ body weight) from P3 to P5. The proliferative EdU+ SCs were stained with the Click‐it EdU imaging kit (Invitrogen, C10337) after blocking. *Lgr5* ^EGFP‐CreER/+^ mice were used as control group and treated in the same manner.

### Lineage Tracing of Lgr5+ Progenitors in vivo

In order to lineage trace Lgr5+ progenitors in the cochlea, two kinds of triple‐positive mice (*Lgr5* ^EGFP‐CreER/+^ *Serpine2* ^loxp/loxp^Rosa26‐*tdTomato* mice and *Lgr5* ^EGFP‐CreER/+^ *Serpine2* ^loxp/+^Rosa26‐*tdTomato* mice) were obtained by crossing Rosa26‐*tdTomato* mice with *Lgr5* ^EGFP‐CreER/+^ *Serpine2* ^loxp/loxp^ mice (Figure S1B, Supporting Information). *Lgr5* ^EGFP‐CreER/+^Rosa26‐*tdTomato* mice were used as control group. To activate the Cre enzyme, all mice were administrated with Tamoxifen (0.075 mg g^−1^ body weight) at P1 via i.p. injection and the cochlea were dissected at P7 for staining and imaging.

### Cochlear Explant Culture and siRNA Transfection

The basilar membranes of P1 *Lgr5* ^EGFP‐CreER/+^Rosa26‐*tdTomato* mice were isolated in cold HBSS with sharp forceps (Dumont, 0209‐5‐PO) and subsequently adhered to cell slides (Biosharp, BS‐09‐RC) coated with Cell‐Tak (Corning, 354240). The tissues were maintained in advanced Dulbecco's modified Eagle medium/F‐12 medium (F12) (Gibco, 12634010) supplemented with N2 (1%, Thermo Fisher, 175020‐48), B‐27 (2%, Thermo Fisher, 17504‐044), b‐FGF (10 ng mL^−1^, Sigma, F0291), EGF (20 ng mL^−1^, Sigma, E9644), IGF (50 ng mL^−1^, Sigma, I8779), and ampicillin (1%, Beyotime, ST008) at 37 °C with 5% CO_2_. 6 h after the culture began, the cochleae were transfected with siRNAs for *Serpine2* (synthesized by GenePharma) by using Lipofectamine 2000 (Thermo Fisher; 11668019) following the manufacturer's instructions for 6 h. 4OH‐tamoxifen (MCE, HY‐16950) was added to the medium at a final concentration of 500 nm to activate the Cre recombinase for 3.5 days all through the culture. The Myosin7a and *tdTomato* double‐positive HCs were examined after 4 days of culture. The siRNA sequence used was shown in Table S4 (Supporting Information).

### AAV‐ie Virus Package and RWM Injection

The most efficient siRNA‐*Serpine2*‐1243 sequence was used to construct the pAAV2‐shRNA target plasmid (Figure S4E, Supporting Information). AAV virus packaging was performed with the assistance of Taitool Company. The procedure was as follows: AAV‐ie was used as the capsid vector Target plasmids (shRNA‐*Serpine2* and control shRNA), capsid plasmids, and helper plasmids were cotransfected into HEK‐293T cells. After culturing for 96 h, the cells and medium were collected, lysed in chloroform, and then harvested for virus isolation and purification by iodixanol gradient ultracentrifugation. P1‐2 *Lgr5* ^EGFP‐CreER/+^Rosa26‐*tdTomato* mice were anesthetized in an ice bath for 2 min. AAV injection was performed only on the left ear of each mouse. The volume of 1.5 µL AAV virus (with a titer of 1E+13 GC mL^−1^) was injected into the RWM by using a glass micropipette (25 µm) controlled by a microelectrode drawing instrument MP500 (RWD). After the injection, the skin incision was closed by using tissue adhesive (3M Vetbond, 1469SB). Mice were placed on the 37 °C warming pad for 10 min and then returned to their mother for continued nursing. After 5 days, the P7 mice were sacrificed to dissect cochlea for immunostaining and statistical analysis.

### 10 × Chromium snRNA Sequencing

snRNA‐seq was performed by using 19 cochleae from *Serpine2* ^loxp/loxp^ mice (control group) and 20 cochleae from *Lgr5* ^EGFP‐CreER/+^ *Serpine2* ^loxp/loxp^ mice (*Serpine2* cOE group) at P7 after tamoxifen injection at P1, with all cochleae from each group pooled into a single sample. The purified nuclei were diluted to a concentration of 700–1200 nuclei µL^−1^ for 10 × Genomics Chromium system. Library preparation was carried out according to the protocol of the 10 × Genomics Chromium Next GEM Single Cell 3ʹ Reagent Kits v3.1 (Cat. No. 1000268). Sequencing was conducted on the Illumina NovaSeq 6000 system by OE Biotech Co., Ltd. The GRCm39 reference genome was used for alignment, and the gene expression matrix was generated and quantified by using the default settings of the 10 × Cell Ranger v3 pipeline (version 8.0.1). The filtered count matrix obtained from the Cell Ranger pipeline was then used for downstream analysis.

### snRNA‐seq Data Quality Control and Cell Annotation

All data analyses were performed in R and Python environments. Preliminary quality control of the data matrices from the two groups obtained via 10 × sequencing was conducted by using Scanpy. Cells with fewer than 500 unique genes, more than 8000 unique molecular identifiers, or a mitochondrial gene proportion greater than 25% were considered low quality and excluded. After initial quality control, 9729 cells from the control group and 11 890 cells from the *Serpine2* cOE group were retained as high‐quality cells. The standard analysis pipeline was then applied in Scanpy for log transformation and normalization. The highly variable genes function was used to select highly variable genes without setting an upper limit. Following dimensionality reduction, the Leiden algorithm was employed for unsupervised cell clustering, and the clustering results were visualized using UMAP. Based on the experimental design, cell populations not included in the subsequent analysis (such as SGNs, glial cells, and complex or unclassifiable cell populations) were removed and the UMAP was reconstructed. In the end, 5846 cells from the control group and 7725 cells from the *Serpine2* cOE group were retained for further analysis. SCs (PC, DC, HeC) and HCs were extracted and reclustering was performed after dimensionality reduction. Unique marker expression was identified in certain HC subpopulations in the *Serpine2* cOE group, including high level of Lgr5+ and expression of some SC markers. These subpopulations were thought to represent transdifferentiation processes or newly generated HC subtypes, termed iHCs.

### Monocle Analysis

HCs and SCs from the *Serpine2* cOE group were converted to loom format for use in the R environment, and Monocle2 (v.2.22.0) was used for pseudotime analysis. Monocle's advanced algorithms allowed the construction of dynamic trajectories of cell differentiation from single‐cell data, which was essential for studying the differentiation of supporting cells into HCs. Using the newCellDataSet function, the object was constructed, selecting genes expressed in at least five cells, and differential gene testing was performed with the differentialGeneTest function. Gene sets with *q*‐values ranging from 1e−60 to 1e−150 were selected for trajectory computation (by using the reduceDimension function with parameters max_components = 2 and method = “DDRTree”). Finally, cell positions along the trajectory were sorted using the orderCells function, and the results were visualized by using the plot_cell_trajectory function.

### Gene Enrichment Analysis and AUC Pathway Scoring

Target cell populations (HCs, DCs, PCs, HeCs, IBCs) were first extracted from both the control and *Serpine2* cOE groups. The rank_genes_groups function in Scanpy was used to perform differential gene ranking for Lgr5+ cells in both groups. Gene sets with significant *p*‐values (log2FC > 0, *p* < 0.05) were selected and subjected to enrichment analysis (including GO and KEGG) by using the Metascape platform (https://metascape.org/gp). Additionally, the score_genes method was employed to assess the activity of relevant pathways such as Wnt, Notch, SHH, cell cycle, and TGF‐β in both groups of cells. The results were visualized by using the pl.violin function.

### Data Analysis

Each experiment was performed for at least 3 times independently. The letter “*n*” represented the number of experimental repeats in the figures. The statistical significance was determined by using the two‐tailed unpaired Student's *t*‐test in GraphPad Prism 8, and the data were shown as means ± standard errors of the means. Data were considered statistically significant when *p* < 0.05.

## Conflict of Interest

The authors declare no conflict of interest.

## Author Contributions

H.X., J.W., L.H., Y.M., and L.W. contributed equally to this work. R.C. and S.Z. conceived and designed the experiments. H.X., L.H., and L.W. performed most of the experiments. J.W. analyzed the single‐nucleus RNA sequencing data. H.X., L.H., Y.M., and S.Z. contributed to critical discussion and data analysis. H.X., L.H., Y.M., and S.Z. wrote the paper. H.X., Y.L., Z.Y., X.T., X.T., W.T., M.D., and Y.W. validated the article. All authors read and approved the final paper.

## Supporting information



Supporting Information

## Data Availability

The data that support the findings of this study are available from the corresponding author upon reasonable request.
