# Supplementary material for: Conditional Overexpression of Serpine2 Promotes Hair Cell Regeneration from Lgr5+ Progenitors in the Neonatal Mouse Cochlea
Source: Adv Sci (Weinh). 2025 Mar 17;12(18):2412653. doi: 10.1002/advs.202412653 (PMC12079390; doi:10.1002/advs.202412653)
Supplement: Supplementary file 1 — Supporting Information [file ADVS-12-2412653-s001.docx]

**Supporting Information**

**Conditional Overexpression of Serpine2 Promotes Hair Cell Regeneration from Lgr5+ Progenitors in the Neonatal Mouse Cochlea**

*Hairong Xiao*, *Jiheng Wu*, *Lixuan Huang*, *Ying Ma*, *Leilei Wu*, *Yanqin Lin*, *Zixuan Ye*, *Xin Tan*, *Xujun Tang*, *Wei Tong*, *Mingchen Dai*, *Yintao Wang*, *Xia Sheng*, *Renjie Chai*^*^, *Shasha Zhang*^*^

**List of contents for supporting information**

1. **Supplementary method**
2. **Supplementary figures**
3. **Supplementary tables**
4. **Supplementary method**

**1.1 Auditory Brainstem Response (ABR) testing**

American TDT audiometry equipment (RZ6, Tucker‐Davis Technologies) and BioSigRZ software were used for ABR testing. P30 mice were anesthetized with 10 mg/ml pentobarbital (0.01 ml/g body weight, intraperitoneal injection) before ABR testing. The recording electrode was inserted subcutaneously in the median line of the skull, the reference electrode and the ground electrodes were inserted subcutaneously behind the bilateral ears. Tone burst ABRs of 4k, 8k, 12k, 16k, 24k and 32k Hz were used as stimuli in the experiment. The intensity of the stimuli started from the maximum stimulus intensity (90 dB SPL) and gradually decreased with 5 dB interval until no repetitive ABR waveform could be detected.

**1.2** **HEI-OC1 culture and siRNA transfection**

The HEI-OC1 cells were cultured in DMEM medium (Gibco, 11965092) supplemented with FBS (10%, Vivacell, C04001-500), and ampicillin (1%, Beyotime, ST008) at 37 °C with 5% CO_2_. When the cell density reached about 60%, the cells were transfected with siRNAs (GenePharma) by using Lipofectamine 2000 (Thermo Fisher; 11668019) following the manufacturer’s instructions for 6 h. After culturing for 24 h, cells were collected in Trizol reagent to extract RNA for subsequent RT-qPCR verification. The siRNA sequence used are shown in Supplementary Table 4.

1. **Supplementary figures**


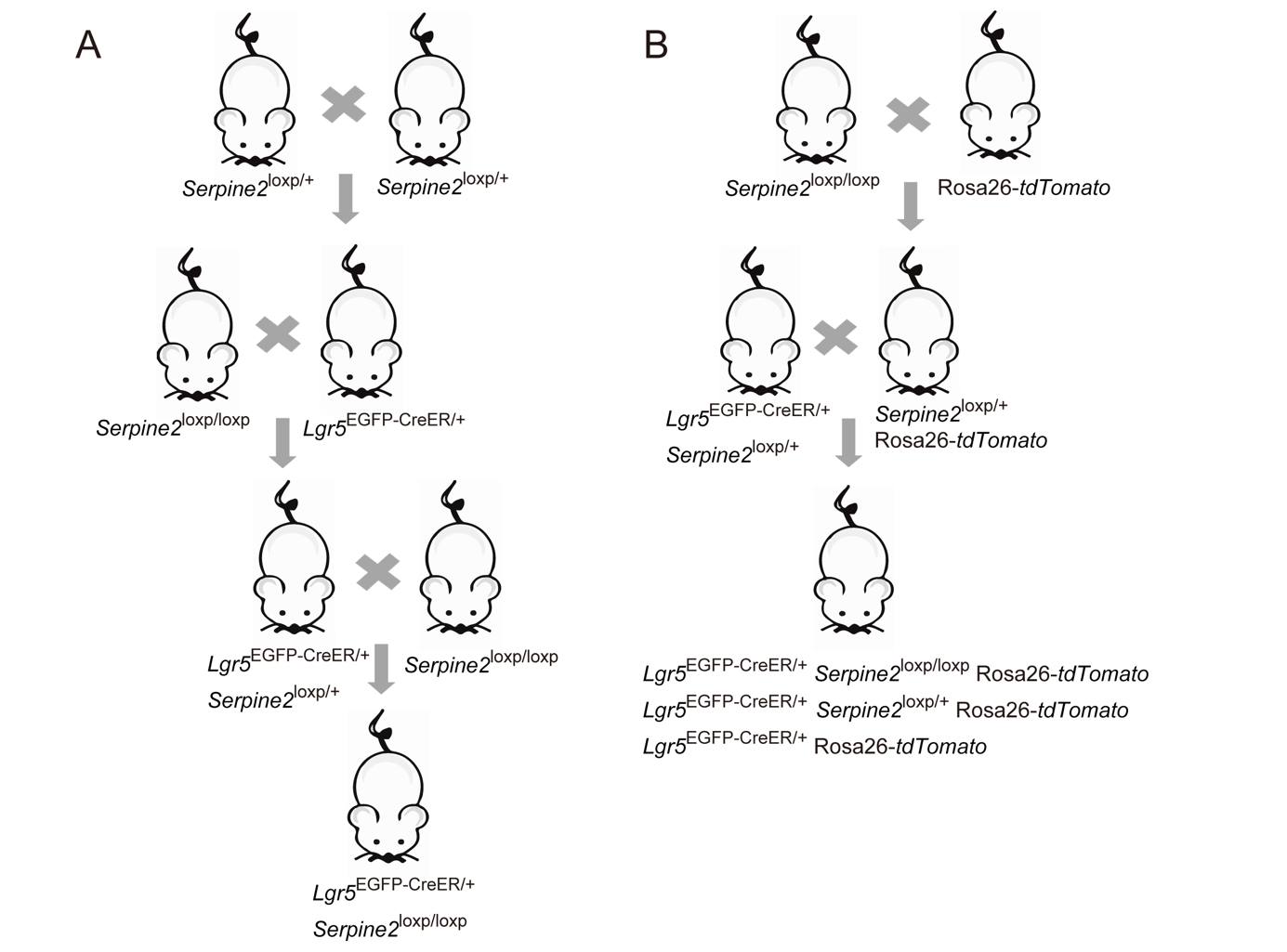


**Figure S1. Schematic diagram of mouse breeding.** **(A)** The flowchart for obtaining *Serpine2* cOE mice (including *Lgr5*^EGFP-CreER/+^*Serpine2*^loxp/+^ mice and *Lgr5*^EGFP-CreER/+^*Serpine2*^loxp/loxp^ mice) for phenotypic exploration. **(B)** The flowchart for obtaining triple-positive mice (*Lgr5*^EGFP-CreER/+^*Serpine2*^loxp/loxp^Rosa26-*tdTomato* and *Lgr5*^EGFP-CreER/+^*Serpine2*^loxp/+^Rosa26-*tdTomato*) for lineage tracing.


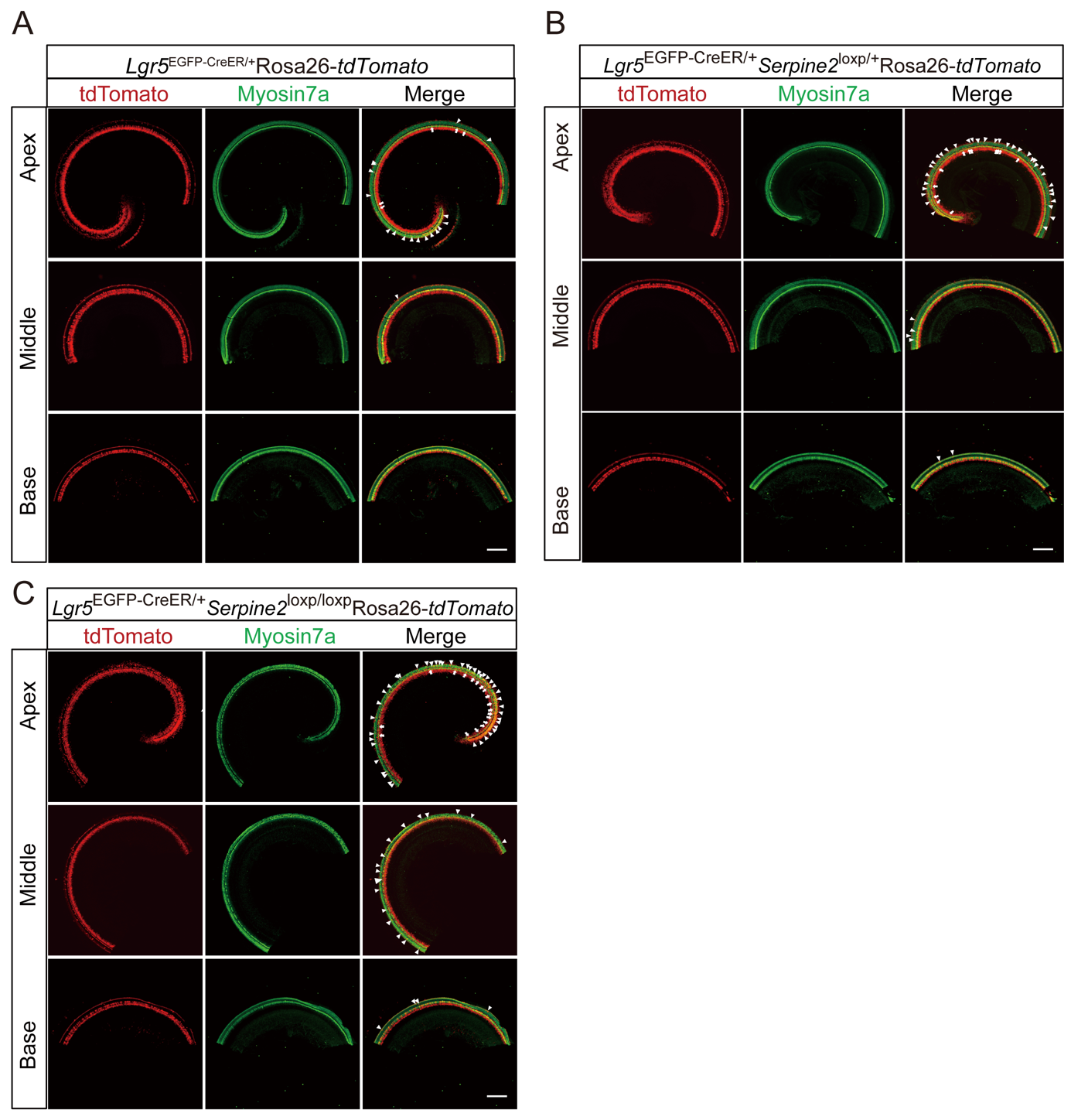


**Figure S2. Lineage tracing of Lgr5+ progenitors in *Serpine2* cOE mice. (A-C)** Lineage tracing images of Lgr5+ progenitors in *Lgr5*^EGFP-CreER/+^Rosa26-*tdTomato* mice (A), *Lgr5*^EGFP-CreER/+^*Serpine2*^loxp/+^Rosa26-*tdTomato* mice (B), and *Lgr5*^EGFP-CreER/+^*Serpine2*^loxp/loxp^Rosa26-*tdTomato* mice (C). tdTomato+ HCs are marked by arrows (IHCs) and arrowheads (OHCs), respectively. Scale bars are 200 µm in (A-C).

**
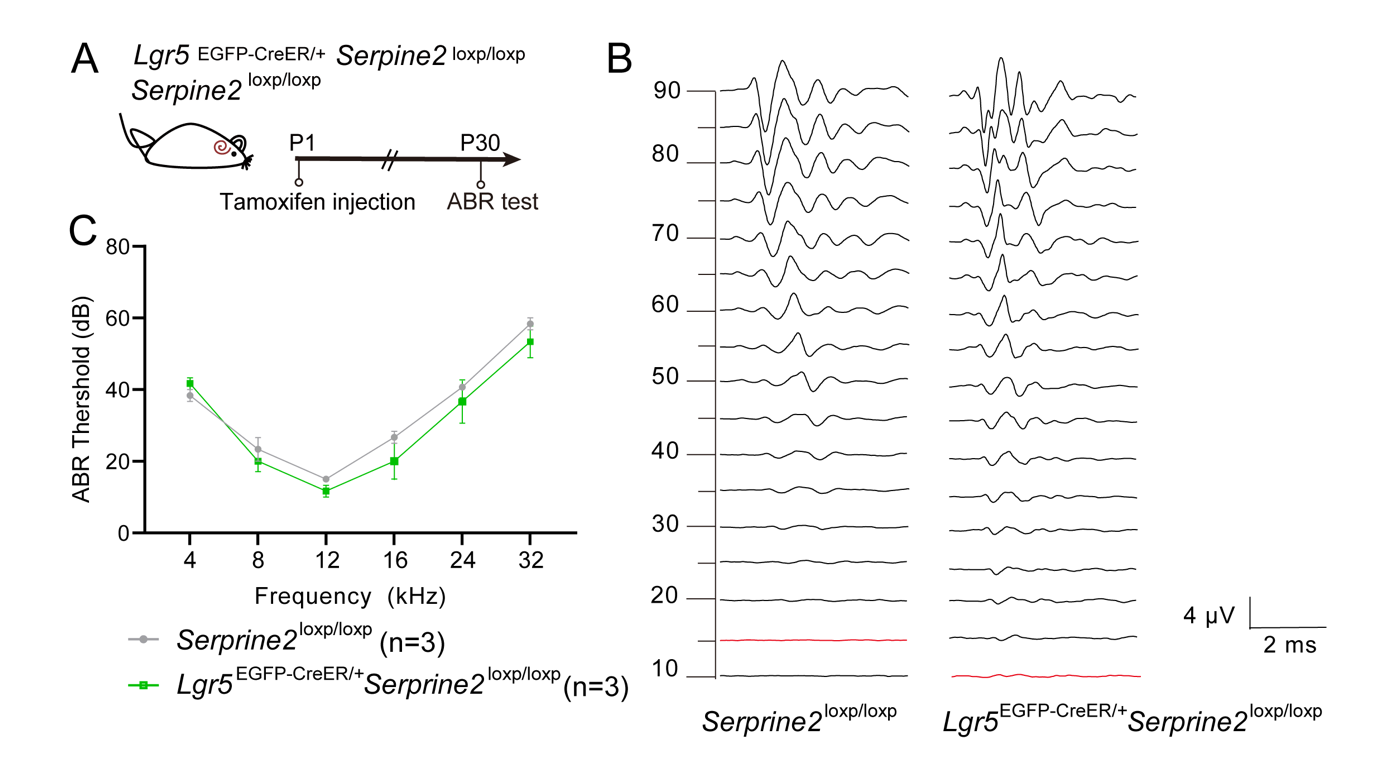
**

**Figure S3. ABR testing of P30** ***Serpine2* cOE mice. (A)** Flow chart of the ABR testing of Serpine2 cOE mice. **(B)** Comparison of the ABR thresholds between *Serpine2* cOE mice and control mice. **(C)** Families of ABR waveforms in 12 kHz were recorded at P30 from the representative *Serpine2* cOE mice and control mice. The red trace indicates the threshold. The scale bar applies to all traces.


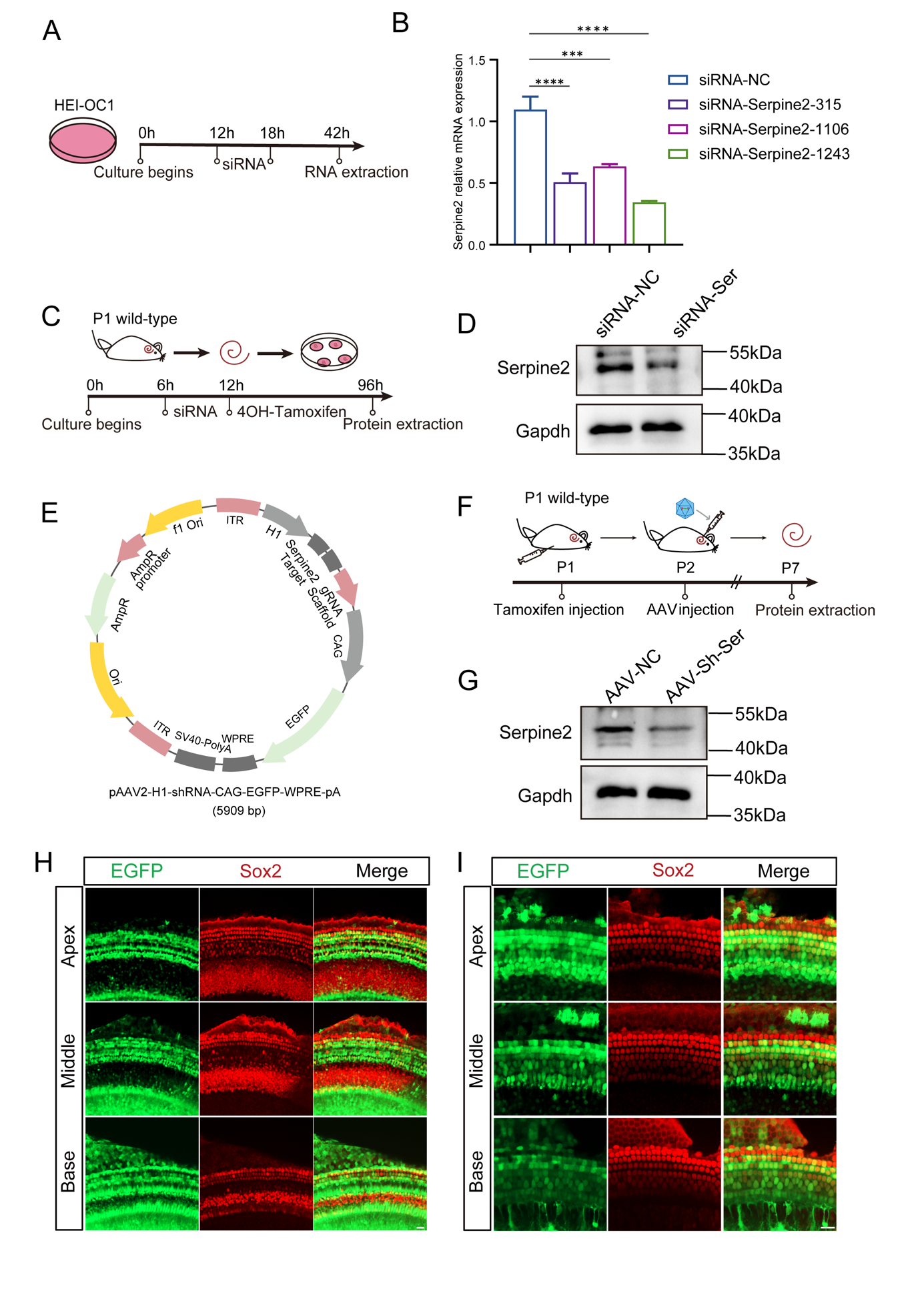


**Figure S4. Knockdown efficiency of *Serpine2* *in vitro, ex vivo* and *in vivo*.** **(A)** Flow chart of siRNA-*Serpine2* knockdown efficiency screening in HEI-OC1 cells *in vitro*. **(B)** Knockdown efficiency of three siRNA-*Serpine2* in HEI-OC1 cell lines by RT-qPCR. **(C)** Flow chart for validation of *Serpine2* knockdown in cochlear explants by siRNA *ex vivo*. **(D)** Knockdown efficiency of siRNA-Ser on cochlear explants by western blotting. **(E)** pAAV2-shRNA-*Serpine2* target plasmid profile. **(F)** Flow chart for validation of *Serpine2* knockdown by AAV *in vivo*. **(G)** Knockdown efficiency of AAV-Sh-Ser *in vivo* by western blotting. **(H-I)** Representative confocal images of AAV-Sh-Ser fluorescence (green) in cochleae transduced at P7 in the mice left ear. Sox2 (red) was used to label SCs. Scale bars are 20 µm in H and I. *** *p* < 0.001, *****p* < 0.0001.


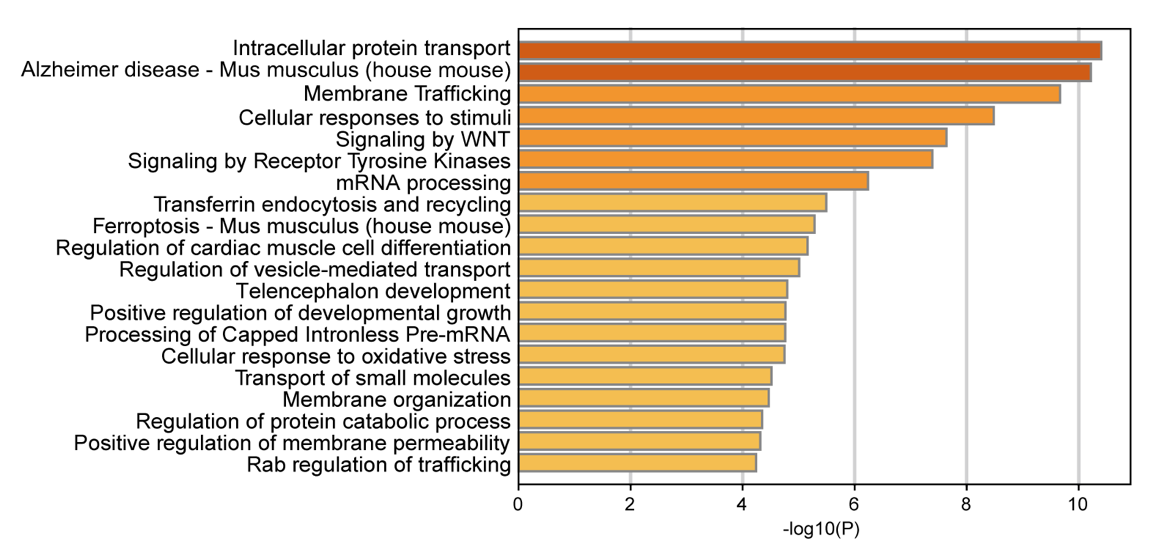


**Figure S5.** Gene enrichment analysis of *Serpine2* cOE mice cochlea via Metascape platform.

Table S1. Quantification of ectopic HCs in P7 *Serpine2*^loxp/+^ mice, *Lgr5*^CreER/+^ *Serpine2*^loxp/loxp^ mice and *Lgr5*^CreER/+^ *Serpine2*^loxp/loxp^ mice. The n represents the number of mice used.

|  | *Serpine2*^loxp/+^  (n=8) | *Lgr5*^CreER/+^  (n=9） | *Lgr5*^CreER/+^ *Serpine2*^loxp/+^ (n=9） | *Lgr5*^CreER/+^ *Serpine2*^loxp/loxp^ (n=8） |
| --- | --- | --- | --- | --- |
| Ectopic OHCs per turn (Apex) | 9.25 ± 3.13 | 2.33 ± 0.97 | 9.22 ± 2.81 | 14.75 ± 1.74 |
| Ectopic OHCs per turn (Middle) | 1.38 ± 0.46 | 1.68 ± 0.73 | 2.67 ± 1.24 | 5.13 ± 1.54 |
| Ectopic OHCs per turn (Base) | 2.38 ± 2.38 | 0.00 ± 0.00 | 1.33 ± 1.33 | 1.13 ± 0.99 |
| Ectopic IHCs per turn (Apex) | 2.25 ± 0.94 | 5.33 ± 0.88 | 5.89 ± 1.36 | 22.38 ± 2.58 |
| Ectopic IHCs per turn (Middle) | 0.63 ± 0.38 | 2.33 ± 0.62 | 3.11 ± 0.84 | 11.63 ± 2.50 |
| Ectopic IHCs per turn (Base) | 0.13 ± 0.13 | 0.33 ± 1.68 | 2.00 ± 0.53 | 8.25 ± 2.06 |
| Total ectopic OHCs per cochlea | 13.00 ± 3.15 | 4.00 ± 1.51 | 13.22 ± 4.06 | 21.00 ± 3.24 |
| Total ectopic IHCs per cochlea | 3.00 ± 1.25 | 8.00 ± 1.39 | 11.00 ± 2.53 | 42.25 ± 5.84 |

Table S2. Sequences of genotyping primers.

| Genotyping primers | Sequence |
| --- | --- |
| *Lgr5* Forward | 5′-CTG CTC TCT GCT CCC AGT CT-3′ |
| *Lgr5* WT Reverse | 5′-ATA CCC CAT CCC TTT TGA GC-3′ |
| *Lgr5* Mut Reverse | 5′-GAA CTT CAG GGT CAG CTT GC-3′ |
| *tdTomato* WT Forward | 5′-AAG GGA GCT GCA GTG GAG TA-3′ |
| *tdTomato* WT Reverse | 5′-CCG AAA ATC TGT GGG AAG TC-3′ |
| *tdTomato* Mut Forward | 5′-GGC ATT AAA GCA GCG TAT CC-3′ |
| *tdTomato* Mut Reverse | 5′-CTG TTC CTG TAC GGC ATG G-3′ |
| *Serpine2* WT Forward | 5′-CAA GCT AAT TTG ACC AGT CAT TGG A-3′ |
| *Serpine2* WT Reverse | 5′-GCA GAC ACC CAG GAT AAG TG-3′ |
| *Serpine2* Mut Forward | 5′-GCA TCG ATA CCG TCG ACC TC-3′ |
| *Serpine2* Mut Reverse | 5′-GCA GAC ACC CAG GAT AAG TG-3′ |

Table S3. Sequences of RT-qPCR primers.

| RT-qPCR primers | Sequence |
| --- | --- |
| *β-actin* Forward | 5′ -ACG GCC AGG TCA TCA CTA TTG- 3′ |
| *β-actin* Reverse | 5′ -AGG GGC CGG ACT CAT CGT A- 3′ |
| *Serpine2* Forward | 5′ -CAC ATG GGA TCG CGT CCA TC- 3′ |
| *Serpine2* Reverse  *Atoh1* Forward  *Atoh1* Reverse  *Gfi1* Forward  *Gfi1* Reverse  *Pou4f3* Forward  *Pou4f3* Reverse  *Gli1* Forward  *Gli1* Reverse  *Gli2* Forward  *Gli2* Reverse  *Gli3* Forward  *Gli3* Reverse  *Hhip* Forward  *Hhip* Reverse  *Ptch1* Forward  *Ptch1* Reverse  *Shh* Forward  *Shh* Reverse  *Smo* Forward  *Smo* Reverse  *Spop* Forward  *Spop* Reverse  *Sufu* Forward  *Sufu* Reverse  *Apc* Forward  *Apc* Reverse  *Axin* Forward  *Axin* Reverse  *Frizzled10* Forward  *Frizzled10* Reverse  *Frizzled9* Forward  *Frizzled9* Reverse  *Gsk3b* Forward  *Gsk3b* Reverse  *Lgr5* Forward  *Lgr5* Reverse  *Wnt16* Forward  *Wnt16* Reverse  *Wnt5a* Forward  *Wnt5a* Reverse  *Wnt6* Forward  *Wnt6* Reverse | 5′ -CAG CAC TTT ACC AAC TCC GTT TA- 3′  5′ -GAG TGG GCT GAG GTA AAA GAG T- 3′  5′ -GGT CGG TGC TAT CCA GGA G- 3′  5′ -GCC TCA GAT GAC CAG GGG A- 3′  5′ -TGT TTG GAC CCT CGG ATA CTC- 3′  5′ -ATG CGC CGA GTT TGT CTC C- 3′  5′ -GGG CTT GAA CGG ATG GTT CT- 3′  5′ -CCA AGC CAA CTT TAT GTC AGG G- 3′  5′ -AGC CCG CTT CTT TGT TAA TTT GA- 3′  5′ -CAA CGC CTA CTC TCC CAG AC- 3′  5′ -GAG CCT TGA TGT ACT GTA CCA C- 3′  5′ -CAC AGC TCT ACG GCG ACT G- 3′  5′ -CTG CAT AGT GAT TGC GTT TCT TC- 3′  5′ -TGA AGA TGC TCT CGT TTA AGC TG- 3′  5′ -CCA CCA CAC AGG ATC TCT CC- 3′  5′ -AAA GAA CTG CGG CAA GTT TTT G- 3′  5′ -CTT CTC CTA TCT TCT GAC GGG- 3′  5′ -AAA GCT GAC CCC TTT AGC CTA- 3′  5′ -TTC GGA GTT TCT TGT GAT CTT CC- 3′  5′ -GAG CGT AGC TTC CGG GAC TA- 3′  5′ -CTG GGC CGA TTC TTG ATC TCA- 3′  5′ -CCA CCT CCG GCA GAA ATG TC- 3′  5′ -CCT CCC GGC AAA AAC TAA AGT- 3′  5′ -GGG ACT GCA CGC CAT CTA C- 3′  5′ -TTG ACG ATA GCG GTA ACC TGG- 3′  5′ -AAA TCC TTC CAT AGT CTT CCC CA- 3′  5′ -TGC TCC TGG TGT TTC ATT ATA GC- 3′  5′ -CTC CAA GCA GAG GAC AAA ATC A- 3′  5′ -GGA TGG GTT CCC CAC AGA AAT A- 3′  5′ -CAT GCC CAA CCT GAT GGG TC- 3′  5′ -GCC ACC TGA ATT TGA ACT GCT C- 3′  5′ -GTG CCA AGC GAT GGA GAT CC- 3′  5′ -GCG TAG AGC GAG CAG AAG AA- 3′  5′ -TGG CAG CAA GGT AAC CAC AG- 3′  5′ -CGG TTC TTA AAT CGC TTG TCC TG- 3′  5′ -AAG GGT GCT GTG TAC TGG AC- 3′  5′ -AGA AGA GAA CCT TAC GGG ACG- 3′  5′ -CAG GGC AAC TGG ATG TGG TT- 3′  5′ -CTA GGC AGC AGG TAC GGT T- 3′  5′ -CAA CTG GCA GGA CTT TCT CAA- 3′  5′ -CAT CTC CGA TGC CGG AAC T- 3′  5′ -GCA AGA CTG GGG GTT CGA G- 3′  5′ -CCT GAC AAC CAC ACT GTA GGA G- 3′ |

Table S4. Sequences of siRNA for *Serpine2*.

| siRNA | Sequence |
| --- | --- |
| NC sense | 5′ -UUC UCC GAA CGU GUC ACG UTT -3′ |
| NC antisense  *Serpine2*-315 sense  *Serpine2*-315 antisense  *Serpine2*-1106 sense  *Serpine2*-1106 antisense | 5′ -ACG UGA CAC GUU CGG AGA ATT -3′  5′ -GCC UCA UGA GAA CGU UGU UTT-3′  5′ -AAC AAC GUU CUC AUG AGG CTT-3′  5′ -CCC UUG GCA UUA CUG AGA UTT-3′  5′ -AUC UCA GUA AUG CCA AGG GTT-3′ |
| *Serpine2*-1243 sense | 5′ -GCA ACA ACU GCA AUC CUA ATT -3′ |
| *Serpine2*-1243 antisense | 5′ -UUA GGA UUG CAG UUG UUG CTT -3′ |

Table S5. The number and proportion of distinct cell types.

| Cell type | Control group | Percentage | *Serpine2* cOE group | Percentage |
| --- | --- | --- | --- | --- |
| TBC | 3366 | 57.58% | 3407 | 44.10% |
| KO | 585 | 10.01% | 1424 | 18.43% |
| RMC | 572 | 9.78% | 913 | 11.82% |
| OSC | 393 | 6.72% | 289 | 3.74% |
| IPhC | 217 | 3.71% | 335 | 4.34% |
| HeC | 119 | 2.04% | 202 | 2.61% |
| PC/DC | 119 | 2.04% | 59 (PC), 135 (DC) | 2.58% |
| IDC | 95 | 1.63% | 307 | 3.97% |
| IBC | 37 | 0.63% | 97 | 1.26% |
| OHC | 309 | 5.29% | 453 | 5.86% |
| IHC | 34 | 0.58% | 104 | 1.35% |
| HC | 343 | 5.87% | 557 | 7.21% |
| Total | 5846 |  | 7725 |  |
